# Hierarchical regulation of the genome: global changes in nucleosome organization potentiate genome response

**DOI:** 10.18632/oncotarget.6841

**Published:** 2016-01-07

**Authors:** Brittany S. Sexton, Brooke R. Druliner, Daniel L. Vera, Denis Avey, Fanxiu Zhu, Jonathan H. Dennis

**Affiliations:** ^1^ Department of Biological Science, The Florida State University, Tallahassee, FL, United States of America; ^2^ Current address: Massachusetts General Hospital Cancer Center and Department of Medicine, Harvard Medical School, Charlestown, MA, United States of America; ^3^ Current address: Division of Gastroenterology and Hepatology, Mayo Clinic, Rochester, MN, United States of America; ^4^ The Center for Genomics and Personalized Medicine The Florida State University, Tallahassee, FL, United States of America; ^5^ Institute of Molecular Biophysics, The Florida State University, Tallahassee, FL, United States of America

**Keywords:** nucleosome, chromatin, iSLK.219, KSHV, next generation sequencing, Chromosome Section

## Abstract

Nucleosome occupancy is critically important in regulating access to the eukaryotic genome. Few studies in human cells have measured genome-wide nucleosome distributions at high temporal resolution during a response to a common stimulus. We measured nucleosome distributions at high temporal resolution following Kaposi's-sarcoma-associated herpesvirus (KSHV) reactivation using our newly developed mTSS-seq technology, which maps nucleosome distribution at the transcription start sites (TSS) of all human genes. Nucleosomes underwent widespread changes in organization 24 hours after KSHV reactivation and returned to their basal nucleosomal architecture 48 hours after KSHV reactivation. The widespread changes consisted of an indiscriminate remodeling event resulting in the loss of nucleosome rotational phasing signals. Additionally, one in six TSSs in the human genome possessed nucleosomes that are translationally remodeled. 72% of the loci with translationally remodeled nucleosomes have nucleosomes that moved to positions encoded by the underlying DNA sequence. Finally we demonstrated that these widespread alterations in nucleosomal architecture potentiated regulatory factor binding. These descriptions of nucleosomal architecture changes provide a new framework for understanding the role of chromatin in the genomic response, and have allowed us to propose a hierarchical model for chromatin-based regulation of genome response.

## INTRODUCTION

In eukaryotic cells, DNA is packed into chromatin. The fundamental subunit of chromatin is the nucleosome: approximately 150 base pairs (bp) of DNA wrapped around a histone octamer core [1, 2]. It has been proposed that nucleosomes play a role in genome response by regulating access to underlying DNA sequence [3]. The position, density, and occupancy of nucleosomes are determined by factors acting in *cis*, DNA sequence patterns, and those in *trans*, protein complexes.

It is now clear that *cis*-acting DNA sequence patterns influence nucleosome distributions. Broadly speaking, two approaches have been taken to classify DNA sequences as nucleosome-forming or nucleosome-inhibitory. The first approach involves the identification of generic dinucleotide occurrences that confer a bendability of DNA around the nucleosome [4, 5]. An alternate approach has been to identify more cryptic and sophisticated genetically-encoded signals using models that discriminate between nucleosome-forming and nucleosome-inhibitory DNA sequences [6, 7]. The precise genetically-encoded signals and the extent to which they direct nucleosome position is still a matter of considerable debate [8].

*Trans-*acting protein complexes, such as ATP-dependent chromatin remodelers, reposition nucleosomes. This is classically described at the Pho5 promoter, where the RSC chromatin remodeler complex repositions nucleosomes [9, 10]. There are also a handful of examples (including MMTV and IFNB promoters), in which chromatin remodeler-mediated nucleosome redistributions potentiate regulatory factor binding [11, 12]. Recently, yeast studies have used subnucleosomal fragments from Micrococcal nuclease (MNase) digestion to infer regulatory factor binding in the context of nucleosome distribution [13, 14]. However, the relationship between chromatin structure and regulatory factor binding in metazoan genomes needs to be elucidated.

In previous studies and the work presented here, we use Kaposi's-sarcoma-associated herpesvirus (KSHV) as a model system to investigate changes in cellular chromatin architecture. KSHV is a human herpesvirus and the etiological agent of three human cancers [15-17]. Like other herpesviruses, KSHV exhibits two alternative life cycles, a quiescent latent stage and a productive lytic stage, both of which are crucial for KSHV pathogenesis [18, 19]. We have made use of the iSLK.219 cell culture system, because it displays tight control of KSHV latency, but can be efficiently induced by doxycycline to undergo KSHV lytic reactivation [20]. While recent studies have shed light on the chromatin landscape of KSHV during latency and upon reactivation of lytic replication [21-24], we are the first to illuminate the effect(s) of KSHV replication on cellular nucleosome distribution. We previously discovered that nucleosome redistributions are widespread, transient, and driven by the underlying sophisticated DNA-encoded nucleosome position information [25]. This observation provided the opportunity and model system to study nucleosome redistributions in the context of *cis-* and *trans*-acting factors and regulatory factor binding.

Here we report the development of a new targeted MNase-seq technology, mTSS-seq. Using mTSS-seq, we show that KSHV reactivation-induced nucleosome remodeling is apparently an indiscriminate event affecting a majority of nucleosomes at all TSSs. This remodeling resulted in the translational repositioning of nucleosomes at 16% of TSSs in the human genome. Consistent with our previous observations, 72% of the loci with translational repositioning of nucleosomes have nucleosomes that are repositioned to locations directed by sophisticated DNA sequence features. The widespread and DNA-directed translational repositioning of nucleosomes were consistent across disparate cell types, suggesting a common program for genome response. Furthermore, we provide evidence that the widespread nucleosomal architecture alterations likely potentiate regulatory factor binding. These results allow us to propose a new chromatin-based hierarchical model for genome response.

## RESULTS

### Mapping nucleosome distributions following KSHV reactivation using the mTSS-seq technique

**Figure 1:**
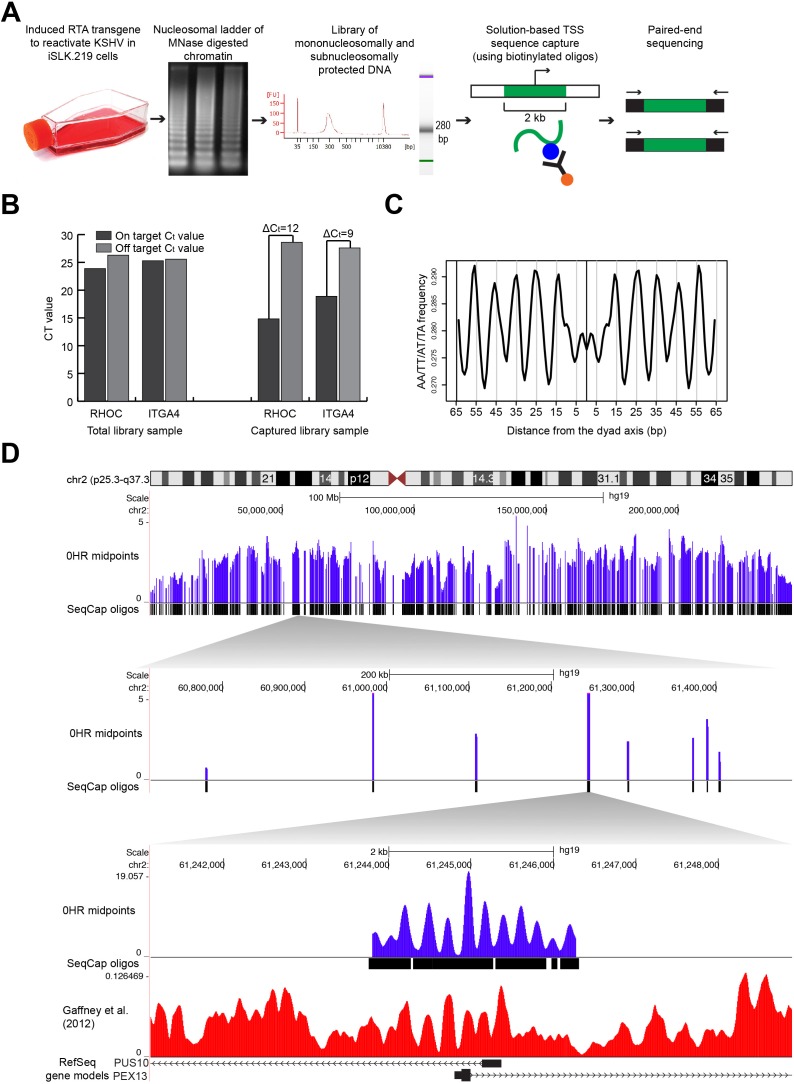
Mapping nucleosome distributions following KSHV reactivation using mTSS-seq technique and mTSS-seq validation **A.** Experimental design for mapping nucleosome distributions following KSHV reactivation using our newly developed mTSS-seq technique. **B.** Validation of mTSS-seq using quantitative PCR for both on-target regions of the genome (within the sequence-capture region, two-kb region centered on a TSS) and off-target regions of the genome (not within the sequence-capture region, outside the two-region centered on a TSS). Quantitative PCR was performed for on- and off-target regions of the genome for both RHOC and ITGA4. The y-axis shows C_t_ values. On average, C_t_ values between the on- and off-targets of the sequence-captured libraries differ by 10.5 cycles. **C.** The periodic occurrence of AA/TT/AT/TA dinucleotides was calculated for all nucleosomal-sized fragments (147-148 bp) at the 0 hour time point. The x-axis represents the distance from the dyad axis. The y-axis is the frequency of AA/TT/AT/TA dinucleotides. An A/T-containing dinucleotide periodicity is seen every 10 bp at the 0 hour time point. **D.** Alignment of the midpoint fragments (purple) from mTSS-seq to the human genome shown in the UCSC Genome Browser (http://genome.ucsc.edu). Zooming in 5000X on human chromosome 2 to a six-kb window with two-kb of midpoint fragments at 0 hour time point (purple lines), along with the sequence-capture oligos and previously-published human-nucleosome distribution map for cell line GM18508 (red lines, (Gaffney et al., 2012)) at the TSSs of PUS10 and PEX13. The x-axis is genomic location. The y-axis is scaled reads per million.

We mapped nucleosome distribution at high resolution following KSHV reactivation. We doxycycline-induced the RTA gene of rKSHV.219 in the iSLK cell line (iSLK.219), and measured the nucleosome distributions at 0, 6, 12, 24, and 48 hours (Figure 1A; [20, 26]. We mapped nucleosome distributions using our new developed mTSS-seq technology. In this sequence-capture approach, we enriched MNase-cleaved fragments for the two kilobases (kb) surrounding the TSSs of 21,547 human open-reading frames. We verified the success of our sequence-capture approach by quantifying the enrichment of the target sequence in the captured libraries as compared to non-captured libraries using quantitative PCR (Figure 1B). We saw on average a difference in C_t_ values between the on- and off-targets of the sequence-captured libraries of 10.5 cycles, suggesting a 500-fold enrichment of target sequences.

We next wanted to ensure that our nucleosome distribution maps shared common features previously observed for nucleosomes and nucleosome distribution maps. The rotational phasing of DNA around the histone octamer results in an acknowledged 10 bp periodicity of A/T-containing dinucleotides [5]. In order to verify that we were measuring positions of nucleosomally-protected DNA fragments, we calculated the frequency of A/T-containing dinucleotides for nucleosomal-sized DNA fragments. We aligned all nucleosomal-sized fragments, 146-148 bp by midpoints [1, 27] at 0 hours (latent state) and calculated the periodic occurrence of AA/TT/AT/TA dinucleotides (Figure 1C). We confirmed an A/T-containing dinucleotide 10 bp frequency in nucleosomal-sized fragments in the latent state. Finally, we verified our nucleosome distribution maps by comparing our mTSS-seq nucleosome maps to a previously published human nucleosome distribution map [28], which show a high degree of concordance (Figure 1D). These results validate our mTSS-seq-generated nucleosome distribution maps.

### Widespread, transient loss of nucleosome positioning following KSHV reactivation

**Figure 2:**
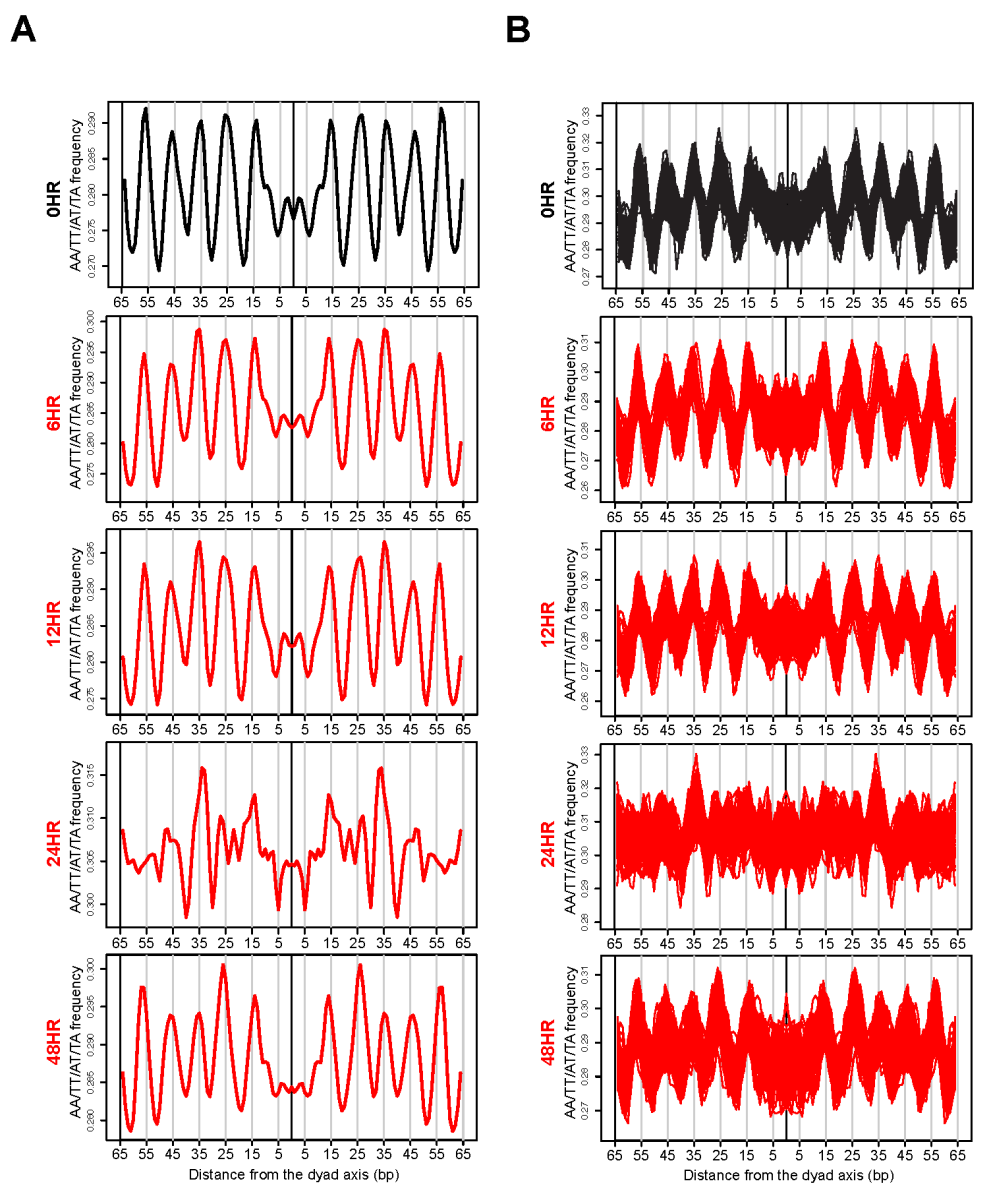
Widespread, transient loss of nucleosome positioning following KSHV reactivation **A.** Periodic occurrence of AA/TT/AT/TA dinucleotides calculated for all nucleosomal-sized fragments (146-148 bp) at the 0 hour (latent state, black line) and 6, 12, 24, and 48 hours after KSHV reactivation (red lines). The x-axis represents the distance from the dyad axis. The y-axis is the frequency of of AA/TT/AT/TA dinucleotides. **B.** Periodic occurrence of an A/T-containing dinucleotides calculated for 3,000 randomized nucleosomal-sized fragments (146-148 bp) 100 times. The 100 iterations were co-plotted for 0 hour (latent state, black line) and 6, 12, 24, and 48 hours after KSHV reactivation (red lines). The x-axis represents the distance from the dyad axis. The y-axis is the frequency of AA/TT/AT/TA dinucleotides.

Our mTSS-seq approach to map nucleosome distributions allowed for a broader assessment of our initial observation that widespread chromatin remodeling is integral to genome response. Our validation experiments demonstrating the 10 bp A/T-containing dinucleotide periodicity in the nucleosome sized fragments at 0 hours, prompted us to investigate the strength of these generic nucleosome position signals at the other time points in the reactivation of KSHV. If remodeling is not targeted, we would hypothesize that the periodic occurrence of AA/TT/AT/TA dinucleotides might be lost at a majority of nucleosome sized fragments during the reactivation of KSHV. We centered and aligned all nucleosomal-sized fragments, 146-148 bp, [1, 2] at 0 hours (latent state) and 6, 12, 24, and 48 hours after KSHV reactivation, and calculated the periodic occurrence of AA/TT/AT/TA dinucleotides. The 10 bp A/T-containing dinucleotide frequency on average was seen at 0, 6, and 12 hours with clear peaks found at 15, 25, 35, 45, and 55 bp from the dyad axis (Figure 2A). The A/T-containing dinucleotide occurrences were aperiodic for the nucleosomal-sized fragments at the 24 hour time point (Figure 2A). The periodicity returned at 48 hours, suggesting that the remodeling event is transient (Figure 2A).

Two possibilities might account for the loss in an A/T-containing dinucleotide periodicity 24 hours after KSHV reactivation. One is that a majority of the nucleosomes shift slightly in either direction, thereby diluting the periodic signal. An alternate explanation would involve a smaller fraction of the nucleosomal-sized fragments cancelling out the periodic signals of the rest. In the first explanation, chromatin remodeling is an indiscriminate event occurring at a majority of all TSSs measured. In the second explanation, chromatin remodeling is a more targeted process. To answer the question of what possibility might account for the loss in an A/T-containing dinucleotide periodicity at the 24 hour time point, we sampled 3,000 random nucleosomal-sized fragments 100 times and co-plotted all 100 iterations of the periodic occurrence of an A/T-containing dinucleotide at each time point after KSHV reactivation (Figure 2B). An A/T-containing dinucleotide periodicity was retained at 0, 6, 12, and 48 hour time points and was lost at the 24 hour time point (Figure 2B). This analysis argues for a majority of nucleosomal-sized fragments shifting in either direction in an indiscriminate, widespread remodeling event. These results suggest that there is a loss of nucleosome rotational phasing signals after KSHV reactivation. We next investigated the consequences of this indiscriminate remodeling event on translational nucleosome repositioning following KSHV reactivation.

### Widespread, transient translational nucleosome repositioning following KSHV reactivation

**Figure 3:**
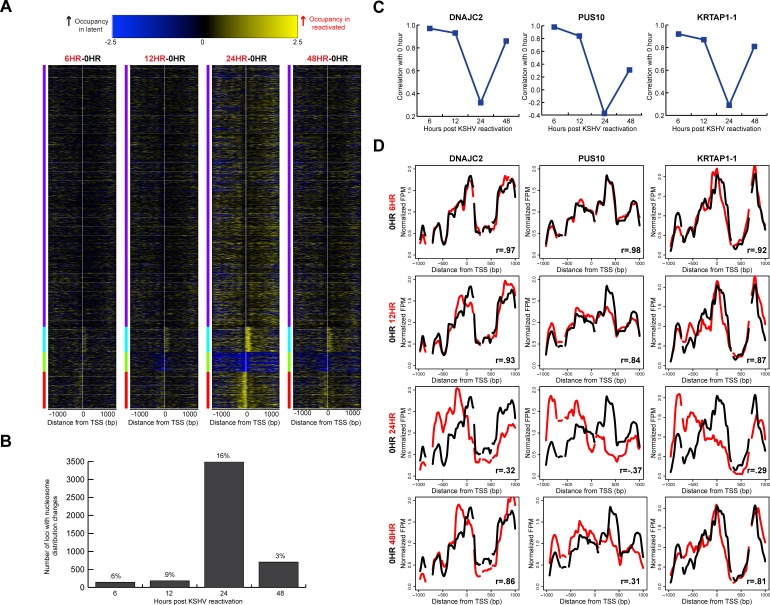
Widespread, transient translational nucleosome repositioning following KSHV reactivation **A.** Heat maps showing differences in nucleosome distributions between 0 hour (latent state) and 6, 12, 24, and 48 hours after KSHV reactivation, sorted on k-means cluster of four. The y-axis is genes of all human open reading frames. The x-axis is two-kb centered on the TSSs of all human open reading frames. The blue within the heat map indicates increased midpoints in the latent stat, and yellow within the heat map indicates increased midpoints in the reactivated time point. **B.** Using a correlation threshold (*r* = 0.7) between the latent state and reactivated states, TSSs with changes in nucleosome distributions were classified for 6, 12, 24, and 48 hours after KSHV reactivation. Approximately one in six TSSs had nucleosome redistributions at the 24 hour time point. **C.** Nucleosome distributions correlations between 0 hours and reactivated state time points for DNAJC2, PUS10, and KRTAP1-1. **D.** Nucleosome distributions of the latent (black line) and reactivated (red lines) KSHV time points for DNAJC2, PUS10, and KRTAP1-1. The x-axis represents genomic position, showing two-kb centered on a TSS. The y-axis is the normalized reads per million. The greatest nucleosome redistributions occur between the latent state and 24 hour time point.

We wanted to know whether the indiscriminate remodeling events were temporally linked with translational repositioning. In our previous study, we observed that 49% of the 472 immunity-related TSSs tested displayed transient translational nucleosome repositioning [25]. We wanted to see if these changes in translational nucleosome positioning were limited to select set of TSSs, or if these nucleosomal architecture alterations were truly a widespread event. We analyzed nucleosome distributions at approximately 21,000 TSSs during KSHV reactivation. We calculated a difference map comparing nucleosome distributions at each TSS between the latent state and at each time point following KSHV reactivation to gain an understanding of the differences in chromatin architecture on a per-locus basis (Figure 3A). Few differences were observed between the latent state and 6, 12, and 48 hours after KSHV reactivation. However, the architecture at 24 hours after KSHV reactivation showed the most differences in nucleosome distribution. These nucleosome redistribution events include increased nucleosome occupancy at the +1 nucleosome at 1,623 TSSs at the 24 hour time point compared to the latent state; 2,307 TSSs with increased nucleosome occupancy at the −1 nucleosome at the 24 hour time point compared to the latent state (Figure 3A); and 1,269 TSSs with decreased nucleosome occupancy at the TSS at the 24 hour time point compared to the latent state (Figure 3A). Genes with loss of nucleosome occupancy at the TSS at the 24 hour time point and were enriched for biological processes including positive regulation of the immune response (p-value=5.68 × 10^−4^) and detection of stimulus (p-value=4.03 × 10^−16^; [29, 30]).

We next wanted to quantify the nucleosome distribution changes at each TSS. We calculated a Pearson correlation coefficient for each TSS between the latent state and the reactivated time points. Using a correlation threshold of r =0.7, we classified TSSs as those with changes in nucleosome distributions (r<0.7, Supplementary Table 1), and those without changes in nucleosome distributions after KSHV reactivation (r ≥ 0.7). We observed that approximately 1 in 6 TSSs (3,474 TSSs) show changes in nucleosome distributions at the 24 hour time point (Figure 3B). By the 48 hour time point, 85% of TSSs with nucleosome redistributions at the 24 hour time point returned to the basal nucleosome architecture (Figure 3B). We were interested in understanding whether the genes whose TSSs had altered nucleosome distributions were enriched for any biological process. We found that the genes with nucleosome redistributions at the 24 hour time point were significantly enriched for biological processes including the detection of stimulus (p-value=2.69 × 10^−42^) and cell surface receptor signaling pathway (p-value=4.94 × 10^−8^; [29, 30]).

We next wanted to analyze the changes in nucleosome distribution on a per-locus basis. We plotted nucleosome distributions for each reactivated time point compared to the latent state. Three exemplar TSSs (DNAJC2, PUS10, and KRTAP1-1) with their corresponding correlation coefficients are shown in Figure 3C-3D. The individual TSS plots show loss of nucleosomal occupancy in some regions at the 24 hour time point and gain in other regions, suggesting translational repositioning of nucleosomes. An exemplar TSS with no changes in nucleosome distribution is shown in Supplementary Figure 1. These results affirmed and extended the observation of widespread, transient translational nucleosome repositioning during KSHV reactivation at all human TSSs.

### Translational nucleosome repositioning is driven by sophisticated DNA-encoded nucleosome position information

**Figure 4:**
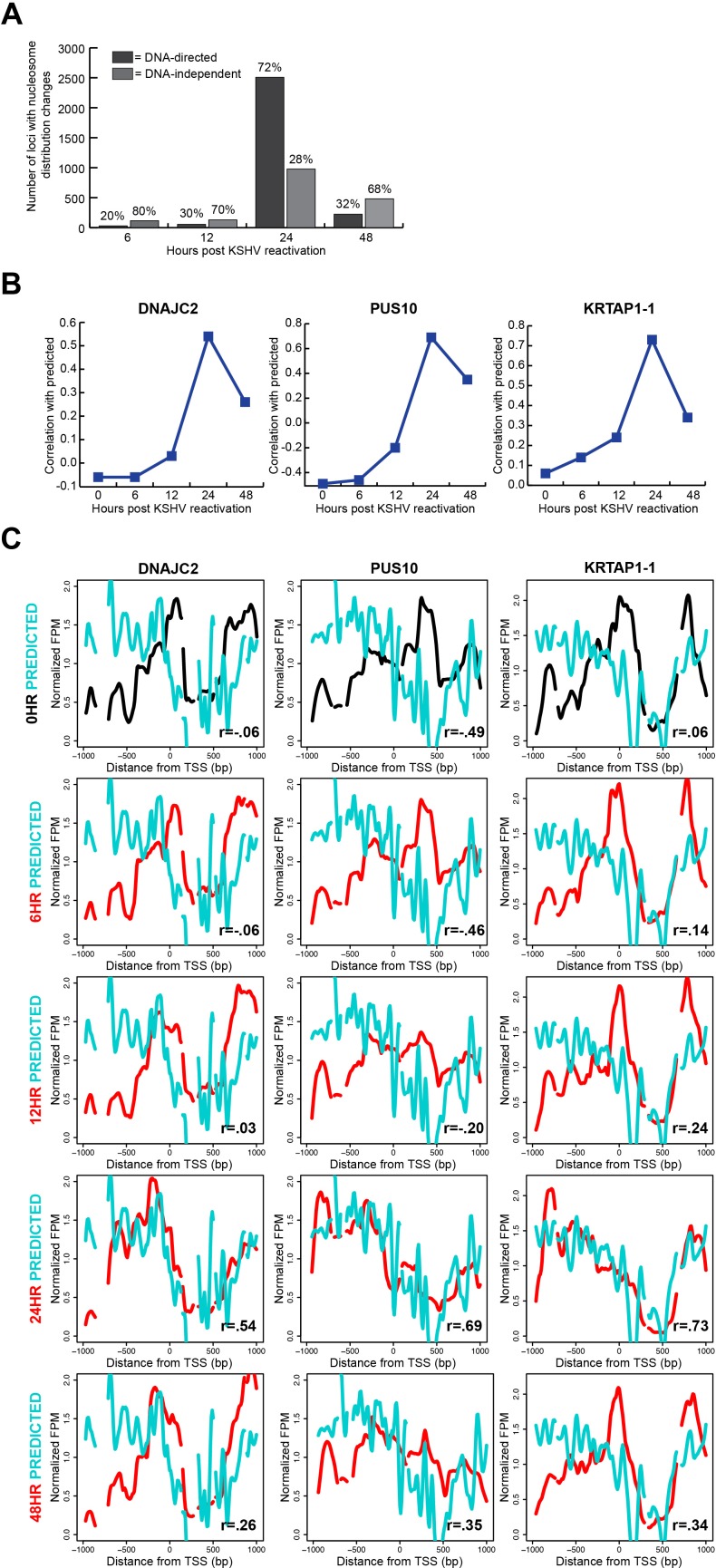
Translational nucleosome repositioning is driven by sophisticated DNA-encoded nucleosome position information **A**. Relative proportions of TSSs with nucleosome redistributions at 6, 12, 24, and 48 hours after KSHV reactivation, classified as DNA-directed or DNA-independent. 72% of TSSs with nucleosome redistributions are DNA-directed at the 24 hour time point. **B**. Correlation values of the 0 hour (latent state) and the reactivated state's time point to the predicted at the same three loci previously shown in figure 3D: DNAJC2, PUS10, and KRTAP1-1. **C**. Predicted (cyan line), latent state (black line), and reactivated state time points (red lines) nucleosome distributions for the same three genes previously shown in figure 3D: DNAJC2, PUS10, and KRTAP1-1. The nucleosome redistributions at the 24 hour time point correlate most strongly with the predicted model

We had previously shown at a limited number of TSSs that a majority of nucleosome redistributions were influenced by the underlying DNA sequence [25]. With this comprehensive study, we were interested in understanding the global nature of the DNA-directed response. We compared a validated model of DNA-directed nucleosome occupancy [6] to our measured nucleosome occupancy in the latent state and each reactivated state's timepoints. In this comparison, we calculated a Pearson correlation coefficient for each TSS with nucleosome redistributions and determined the time point at which the nucleosome redistributions agreed best with the computational model. We found that 72% of the 3,474 loci with nucleosome redistributions at the 24 hour time point occupied positions predicted by the underlying DNA sequence (Figure 4A; Supplementary Table 1). Exemplar loci, previously shown in Figure 3D, demonstrate the relationship between the DNA sequence-preferred positions and measured nucleosome occupancy at each time point (Figure 4B-4C). These results are consistent with our previous limited-scope study, and suggest a substantial role for DNA sequence in directing chromatin-based genome response.

Our classification of the TSSs with translational nucleosome repositioning as DNA-directed or DNA-independent provided the opportunity for us to investigate any overarching features of TSS architecture associated with each class. On average, DNA-directed loci at the 24 hour time point had higher relative occupancy upstream and downstream of the TSS, and lower occupancy at the TSS when compared to those at the 0 hour time point (Supplementary Figure 2, left column). The DNA-independent loci on average had lower nucleosome occupancy than the DNA-directed loci (Supplementary Figure 2, right column). The average patterns of nucleosome occupancy for the DNA-independent loci showed a modest decrease in occupancy at the 24 hour time point. Likewise, the predicted nucleosome occupancy for these loci does not indicate strong nucleosome-forming or -inhibitory DNA signals. We interpret these results to mean that the DNA-independent loci have a less defined chromatin structure as indicated by a lack of strong predicted or measured nucleosome occupancy signals. These aggregate DNA-directed and DNA-independent profiles suggest two distinct modes for making genomic DNA available.

### Nucleosome redistributions driven by sophisticated DNA-encoded nucleosome position information are consistent across disparate cell types

**Figure 5:**
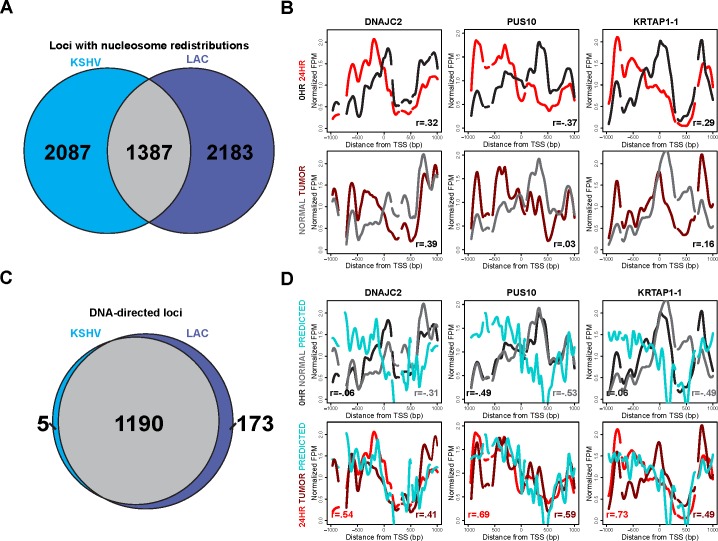
Nucleosome redistributions driven by sophisticated DNA-encoded nucleosome position information are consistent across disparate cell types **A.** Venn diagram showing the shared and unique TSSs with changes in nucleosome redistributions, at 24 hours after KSHV reactivation (left side, blue) and in the lung adenocarcinoma (LAC) low-grade tumor sample (right side, purple). We found that 40% of the 3,474 loci with nucleosome redistributions at the 24 hour time point agree with those in the LAC low-grade tumor sample. **B.** Nucleosome distributions of the latent (black line), 24 hours after KSHV reactivation (red line), normal LAC sample (gray line), and low-grade LAC tumor sample (dark red line) shown in the same three TSSs previously shown: DNAJC2, PUS10, and KRTAP1-1. **C.** Venn diagram showing the shared and unique TSSs classified as DNA-directed, subset of the TSSs with shared nucleosome redistributions from Figure 5A, for both 24 hours after KSHV reactivation (left side, blue) and the LAC low-grade tumor sample (right side, purple). 85% of the loci with nucleosome redistributions, shared between 24 hours after KSHV reactivation and LAC low-grade tumor sample, are DNA-directed. **D.** Latent state (black line), 24 hours after KSHV reactivation (red line), normal LAC sample (gray line), low-grade LAC sample (dark red line), and predicted (cyan line) nucleosome distributions for the same three TSSs previously shown in figure 3D: DNAJC2, PUS10, and KRTAP1-1.

Given the DNA-directed nature of these nucleosome redistributions at the 24 hour time point, we next wanted to determine whether this response was shared amongst disparate cell types. Druliner et al. demonstrated widespread and DNA-directed nucleosome redistributions in low-grade LAC tumor samples. We compared the KSHV latent state and 24 hour reactivated time point with the respective normal sample and low-grade LAC tumor sample's nucleosome redistributions. 40% of the TSSs identified as having nucleosome redistributions at the 24 hour time point overlap with those identified as nucleosome redistributions at low-grade LAC samples (Figure 5A). We next wanted to see if these nucleosome distribution changes were consistent at individual TSSs. Nucleosome distribution changes between the KSHV latent state and the 24 hour reactivated state compared with the nucleosome redistributions between the normal sample and the low-grade LAC sample show strong agreement at exemplar loci: DNAJC2, PUS10, and KRTAP1-1 (Figure 5B).

The similarities between the nucleosome redistributions led us to investigate whether the DNA-directed nature of these nucleosome redistributions following KSHV reactivation was shared between disparate cell types. Of the 1387 TSSs with shared nucleosome redistributions (Figure 5A) between KSHV reactivation and low-grade LAC sample, 86% were DNA-directed (Figure 5C). These TSSs with nucleosome redistributions, previously shown in Figure 3D, showed agreement with the predicted model and each other (Figure 5D). These results suggest that nucleosomal alterations driven by sophisticated-DNA encoded information may well be a general genomic response shared across disparate cell types.

### Nucleosome architecture alterations following KSHV reactivation potentiate regulatory factor binding

**Figure 6:**
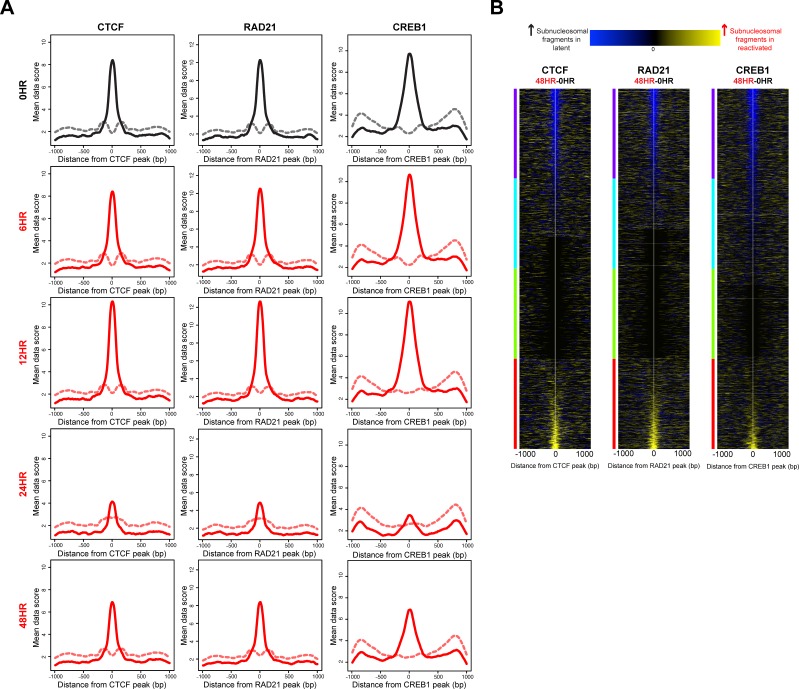
Nucleosome architecture alterations following KSHV reactivation potentiate regulatory factor binding **A.** Histogram of subnucleosomal-sized fragments (< 125 bp, solid line) and nucleosomal-sized fragments (> 125 bp, dashed line) at CTCF, RAD21, and CREB1 binding sites in the latent state (black line) and post-KSHV reactivation time points (red lines). **B.** Heat maps identifying differences in subnucleosomal-sized fragments at CTCF, RAD21, and CREB1 binding sites, between the latent state and 48 hour time point, sorted on max cluster of four. The y-axis is regulatory factor binding sites. The x-axis is two-kb centered on the regulatory factor binding site. The blue within the heat map indicates increased subnucleosomal-sized fragments in the latent stat, and yellow within the heat map indicates increased subnucleosomal-sized fragments in the 48 hour time point.

Given the consensus view that nucleosome distribution regulates access to regulatory factor binding sites [13, 14], we were interested to know whether the nucleosome redistributions at the 24 hour time point potentiated regulatory factor binding. We compared our nucleosome distribution maps with regulatory factor binding events. Using subnucleosomal-sized DNA fragments (<125 bp) from the paired-end sequencing as a surrogate for regulatory factor binding [13, 14, 31], we measured depletion or enrichment of regulatory factor-sized protections at known regulatory factor binding sites, within our sequence-capture regions. These binding sites were identified by chromatin immunoprecipitation (ChIP) in an epithelial cell line similar to the iSLK cell line, A549 [32]. The ENCODE-generated, publically available datasets for 23 ChIPs for the A549 cell line include 18 transcription factors and 5 regulatory factors [33]. We tested each of the A549-derived location maps for differences in subnucleosomal-fragment distributions between the latent state and post-KSHV-reactivation time points. For 22 out of 23 factor binding sites tested, we observed a statistically significant decrease of subnucleosomal-fragments at the 24 hour time point compared to the latent state. No significant difference was observed at the 6 hour or 12 hour time points (Supplementary Figure 3). The subnucleosomal-fragment levels at the 48 hour time point were returning to levels previously seen at the 0 hour time point (Supplementary Figure 3). This phenomenon is shown in the fragment density maps for three exemplar factor binding sites: CTCF, RAD21, and CREB1 (Figure 6A).

We next wanted to know the net change of subnucleosomal-fragments at individual regulatory factor binding sites between the latent and 48 hour time points. To answer this question, we generated a difference map of subnucleosomal-fragments for the 0 hour (latent state) versus the 48 hour time points, to quantify increased or decreased binding at CTCF, RAD21, and CREB1 (Figure 6B). We observed differential binding between the latent state and 48 hour time points. These results indicate that a consequence of the altered chromatin architecture may involve generation of a new regulatory potential for the cell by differential regulatory factor binding.

**Figure 7:**
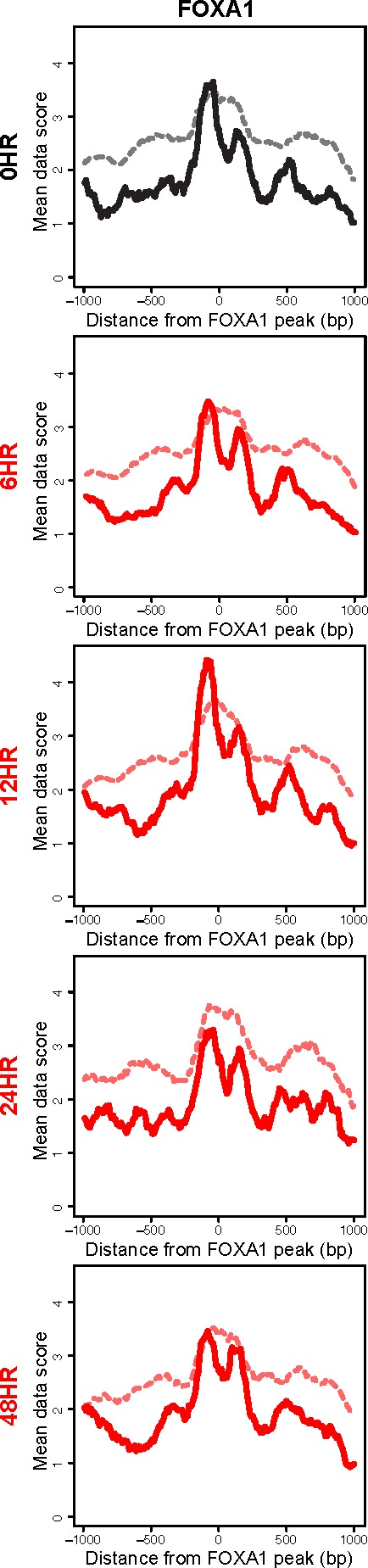
The pioneer factor FOXA1 remains consistently bound following KSHV reactivation Histogram of subnucleosomal-sized fragments (< 125 bp, solid line) and nucleosomal-sized fragments (> 125bp, dashed line) at FOXA1 binding sites in the latent state (black line) and post-KSHV reactivation time points (red lines).

An interesting outlier in this analysis was the FOXA1 transcription factor. FOXA1 is classified as a pioneer transcription factor, belonging to a small family of transcription factors that initially establish capacity for gene expression [34]. These factors have been shown to bind nucleosomally-protected DNA [35]. FOXA1 was the only pioneer transcription factor that we analyzed, and the only factor to show no significant difference in subnucleosomal fragments between the latent state, the 24 hour and the 48 hour time points (Figure 7 and Supplementary Figure 3). In fact, the subnucleosomal fragments show only modest changes throughout KSHV reactivation. Interestingly, consistent with the ability of these factors to bind nucleosomal DNA, the nucleosomal fragments (>130 bp) were unchanged at all time points and were centered on the FOXA1 binding site (Figure 7). Two notable interpretations result from the anomalous characteristics of FOXA1 in this analysis. First, these results suggest *bona fide* FOXA binding, as the subnucleosomal-sized fragment peaks are found flanking the bounds of the nucleosomal fragment at FOXA1 binding sites (Figure 7; [35], which lends credence to all other observations of nucleosome- and factor-binding dynamics. Second, the results suggest that this pioneer factor is unaffected by the widespread remodeling event occurring following KSHV reactivation, and indicate an inherent epigenomic stability at FOXA1 binding sites.

## DISCUSSION

In this study we measured nucleosome distributions at high temporal resolution following KSHV reactivation at the two-kb surrounding the TSSs of all human open-reading frames using our newly-developed mTSS-seq technology. Our results suggest a global indiscriminate remodeling event as measured by a loss of rotational phasing characteristics. Along with the more subtle loss of rotational phasing signals, a subset of loci showed widespread changes in translational positioning of nucleosomes. We demonstrate that 72% of the loci with translational repositioning of nucleosomes are driven by sophisticated genetically-encoded DNA sequence features. We suggest that this is a general feature of genomic response, as translational repositioning of nucleosomes is consistent across disparate cell types. Additionally we show that these alterations in nucleosome architecture likely potentiate regulatory factor binding. The alterations in nucleosome architecture after KSHV reactivation are transient and nucleosomes return to their basal architecture. In aggregate, these results support a new chromatin-based hierarchical model for genome response.

Following KSHV reactivation, there is a loss of the acknowledged rotational phasing signals for nucleosomes [5], as measured by the loss of the 10 bp periodicity of A/T-containing dinucleotides. Our data indicate that this global and indiscriminate remodeling of these nucleosomes allows for a robust and efficient genomic response to occur. Consistent with this observation, previous studies have shown that chromatin remodelers are found throughout the genome and are enriched at TSS and enhancers [36-38]. Our interpretation of these results is that the reactivation of KSHV, or other acute responses, results in a comprehensive recruitment of chromatin regulatory factors, leading to a global “genomic vibration.” The manner in which individual loci respond to their regulation is dependent upon the cellular physiology and biochemical state of the chromatin.

Along with the loss of rotational phasing of nucleosomes after KSHV reactivation, there are widespread changes in translational positioning of nucleosomes. These results suggest that specific loci with the appropriate biochemical potential are susceptible to translational repositioning of nucleosomes. The indiscriminant nature of the remodeling event combined with the specificity of translational repositioning is a plausible system to elicit a concerted response in a complex genome. The efficiency of this system is enhanced by a mechanism to direct the translational repositioning by the underlying DNA sequence.

Multiple models of genetically-encoded nucleosome-forming potential have been developed [4, 6, 39, 40]. Broadly, these fall into two classes: (1) those that identify position-specific bendability features, and (2) those that use machine learning to identify features that discriminate between nucleosome-forming and -inhibitory sequences. Unsurprisingly, because these approaches identify different characteristics, they frequently disagree [41]. Our work sheds light on the biological situations that rely on genetically-encoded features that maintain the basal architecture, and those that direct the remodeled state. We found an A/T-containing dinucleotide periodicity at all time points except the remodeled state (24 hour time point). This observation suggests that a genetically-encoded generic bendability feature is responsible for maintaining the basal architecture of the genome. In the remodeled state, however, the nucleosomes lose their A/T-containing dinucleotide periodicity and instead adopt positions identified as nucleosome-forming by algorithms that account for more sophisticated genetically-encoded patterns. Our work does much to reconcile the literature on the role of DNA sequence in maintenance and regulation of nucleosome positions.

It will be important in the future to understand the forces and factors that maintain nucleosome architecture in it's basal state and regenerate it. An appealing group of candidates that might maintain and regenerate the nucleosome architecture in its basal state is the transcriptional machinery. Some of the transcriptional machinery is found dispersed throughout the genome, even though not always associated with active transcription. We propose that transcriptional machinery could play an active role in the maintenance of the basal state's nucleosomal architecture, and in the post-stimulus return to the basal nucleosomal architecture. In such a scenario, it is likely that the remodeling event would lead to altered regulatory factor binding. Likewise, it will be important to delineate the exact causes and consequences of the remodeled state. Our work moves the field forward in this respect by demonstrating that the remodeling of nucleosomes is concomitant with the disassociation of regulatory factors and their subsequent opportunistic re-association with the genome.

The alterations in nucleosome architecture potentiate regulatory factor binding following KSHV reactivation. There is a loss of regulatory factor binding at 24 hours after KSHV reactivation, followed by a new regulatory factor binding landscape at the 48 hour time point. Our interpretation is that this specified response is driven by cell-type-specific regulatory factors binding in a concentration-dependent manner. The timing of these events is regulated by the transient nature of the remodeling event. We propose a genomic “transient intermediate state” defined by widespread and transient remodeling coupled with altered regulatory factor binding.

In the genomic “transient intermediate state” there was a genome-wide indiscriminate remodeling and widespread translational repositioning. We suggest that this represents an efficient strategy for genome response. The genomic “transient intermediate state” allowed for a superset of loci to be made available to drive an appropriate genomic response. These loci might be bound or unbound in a manner appropriate to the cellular physiology and the response. Following the “transient intermediate state,” nucleosomes return to their basal positions. These observations have prompted us to introduce a chromatin-based hierarchical model for genome response.

**Figure 8:**
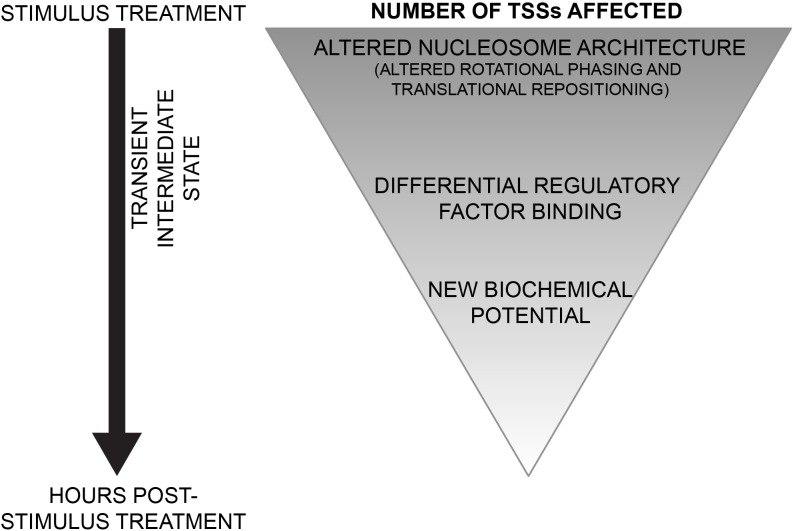
Model for chromatin-based hierarchical regulation of genome response In response to a stimulus, global loss of rotational phasing and widespread translational repositioning of nucleosomes are directed by the underlying sophisticated DNA sequence. These alterations in nucleosomal architecture potentiate regulatory factor binding. The differential regulatory factor binding events drive appropriate genome response and set up a new biochemical potential for cells.

We introduce a new chromatin-based hierarchical model for genome response (Figure 8). In this model, during a response to a stimulus, there is an indiscriminate nucleosome remodeling, as observed by the loss of rotational phasing of nucleosomes. At a superset of loci, widespread and DNA-directed translational repositioning of nucleosomes occurs. These changes in nucleosome architecture potentiate new landscapes of regulatory factor binding. The differential regulatory factor binding events drive appropriate genome response and set up a new biochemical potential for cells. The chromatin-based hierarchical model gives new insight into regulation of genome response.

## MATERIALS AND METHODS

### Cell growth and KSHV reactivation in iSLK.219 cells

Derivative of the iSLK cell line, the iSLK.219 cell line was previously latently infected with the RTA-doxycycline inducible rKSHV.219 virus (Vieira and O'Hearn 2004). The iSLK.219 cells were cultured and maintained according to Myoung and Ganem (2011). Cells were seeded at a density of 5 × 10^6^ cells per 150mm dish, 24 hours prior to the induction of KSHV reactivation in the iSLK.219 cell line. At 24 hours after seeding, the old medium was replaced with DMEM medium containing 1% FBS and a final concentration of doxycycline of 0.2 μg/ml. Cells from the iSLK.219 0, 6, 12, 24, and 48 hours were harvested without added doxycycline. Next, iSLK.219 cells were harvested at 6, 12, 24, and 48 hours after doxycycline addition. The corresponding untreated iSLK.219 cell timepoints showed little nucleosome distribution changes (Supplementary Table 3 and Supplementary Figure 4).

### Cell harvest and nuclei purification

iSLK.219 cell lines were harvested at 2.5 × 10^7^ cells, cross-linked in 1% formaldehyde in PBS, and incubated for 10 min at room temperature. After the 10 min incubation, the cross-linking reaction was stopped by addition of 125 mM glycine. Next, the nuclei were isolated in nucleus isolation buffer containing: 10 mM HEPES at pH 7.8, 2 mM MgOAc_2_, 0.3 M sucrose, 1 mM CaCl_2_, and 1% Nonidet P-40. The nuclei were then pelleted by centrifugation at 1000g for 5 min at 4°C.

### MNase cleavage and purification of mononucleosomal and subnucleosomal DNA

iSLK.219 nuclei were digested for 5 min at 37°C with a titration of MNase: 4 units/mL, 2 units/mL, and 1 unit/mL of MNase (Worthington Biochemical Corp.) in MNase cleavage buffer (4 mM MgCl_2_, 5 mM KCl, 50 mM Tris-Cl (pH 7.4), 1 mM CaCl_2_, 12.5% glycerol). The MNase digestion reactions were stopped with 50 mM EDTA. Next, the protein-DNA crosslinks were reversed by treating the MNase-digested nuclei with 0.2 mg/mL proteinase K and 1% sodium dodecyl sulfate, and incubating overnight at 60°C.

The samples were then run and the nucleosomal ladder was separated on a 2% agarose gel. Following the separation of the DNA fragments, mononucleosomally-sized and subnucleosomal-sized fragments (<200 bp) were isolated from the agarose gel, and the DNA was purified by electroelution. Next, the mononucleosomal- and subnucleosomal-sized fragments for all MNase concentrations were combined for each respective sample. Following the combination of all fragments per sample, DNA was extracted with phenol-chloroform and precipitated with alcohol for 10 min at −20°C. The DNA was then pelleted by centrifugation at 3000g for 10 min at 4°C, and dissolved in TE (0.1 mM EDTA, 10 mM Tris-Cl at pH 8.0).

### Mononucleosomal and subnucleosomal DNA library preparation

Using the NEBNext® Ultra™ DNA Library Prep Kit for Illumina® (NEB #E7370S/L), DNA sequencing libraries were prepared for the mononucleosomally-sized and subnucleosomal-sized fragments for each sample. DNA was end-prepped using NEB Prep enzyme mix, end-repair reaction buffer (10X), and 30 ng of DNA for each samples, then held at 30 degrees Celsius for 30 minutes and then at 65 degrees Celsius for 30 minutes. Adaptors were ligated onto the end-repaired samples by adding NEB Blunt/TA Ligase Master Mix, NEBNext Adaptor for Illumina, and Ligation Enhancer and incubating at 20 degrees Celsius for 15 minutes. The adaptor-ligated DNA was cleaned up using AMPure XP beads to remove any unwanted ligated products.

The universal and indexed sequences were added by PCR using 23 ul of adaptor-ligated DNA fragments, NEBNext High Fidelity 2X PCR Master Mix, index primers provided in NEBNext Multiplex (NEB #E7335, #E7500) Oligos for Illumina, and Universal PCR Primers provided in NEBNext Multiplex (NEB #E7335, #E7500) Oligos for Illumina. Then PCR was done for 8 cycles (not including the initial denaturation and final extension). The adaptor-ligated DNA was cleaned up using AMPure XP beads to remove any unwanted products. The libraries were quality-checked using Agilent 2100 Bioanalyzer High-Sensitivity. Across the libraries, the samples ranged between 200-400bp and there were no adapter or primer dimers.

### Solution-based sequence-capture of DNA fragments within two-kb of all human TSSs

Utilizing the custom-designed Roche Nimblegen SeqCap EZ Library SR, we sequence-captured the previously libraried fragments within the two-kb window surrounding all human TSSs, using the HG19 build. We followed the Roche Nimblegen protocol for the sequence-capture procedure. Following the sequence-capture, we then PCR amplified our sequence-captured fragments using TruSeq primers 1 and 2 (AATGATACGGCG ACCACCGAGA and CAAGCAGAAGACGGCATACGAG, respectively). To determine whether fragments within the two-kb window around all human TSSs were enriched, we performed quantitative PCR for on- and off-target regions of our sequence-captured fragments. The primers used for the quantitative PCR were as follows: on-target primers for RHOC (chr1:113250099-113250499, forward primer: AGATGTCCACCCTCTTGTTCC, and reverse primer: CCAGGGAAGAAAGCGAATTG), off-target primers for RHOC (chr1:113246266-113246422, forward primer: TTGCTGAAGACGATGAGGAG, and reverse primer: CAATCCGAAAGAAGCTGGTG), on-target primers for ITGA4 (chr2:182321015-182321415, forward primer: TATGGCTGTCTCTCTGGTTGC, and reverse primer: AACGCAACACACCTGAACTG), and off-target primers for ITGA4 (chr2:182322923-182323044, forward primer: CAACGCTTCAGTGATCAATCC, and reverse primer: GAGCTGTTCGCACGTCTG).

### Illumina paired-end sequencing and analysis

Using a single lane on an Illumina HiSeq 2500, HiSeq Flow Cell v3, the samples were loaded at 12 pM. The libraries were sequenced using standard Illumina sequencing protocols. Two kits were used: the TruSeq SBS Kit v3 and the TruSeq PE Cluster Kit v3 -cBot – HS. The reads were demultiplexed using the Casava Software, and the library adaptors were removed using the cutadept software [42].

The sequenced fragments were aligned to the HG19 assembly of the human genome using bowtie2 2.1.0 [43]. Using samtools, non-unique and non-paired fragments were removed from the sequenced fragments [44]. The number of sequenced fragments for each sample is denoted in Supplementary Table 4.

Nucleosome distribution maps were determined through BAM files and the use of bedtools 2.17 [45]. Nucleosome distributions were calculated by fragments per million for each bp in the 2kb surrounding each TSS. Midpoints for nucleosome distributions were determined through the calculation of center fragments in 100 bp windows at a 10 bp step in the 2kb surrounding each TSS. Further analysis of the nucleosome distributions was done in the R environement, R 2.15.1 [46], using our lab-developed software, RAGE.

## SUPPLEMENTARY MATERIAL FIGURES AND TABLES


