# Supplementary material for: Hierarchical regulation of the genome: global changes in nucleosome organization potentiate genome response
Source: Oncotarget. 2016 Jan 7;7(6):6460–75. doi: 10.18632/oncotarget.6841 (PMC4872727; doi:10.18632/oncotarget.6841)
Supplement: Supplementary file 1 [file oncotarget-07-6460-s001.pdf]

Hierarchical regulation of the genome: global changes in nucleosome organization potentiate genome response

Supplementary Material

A

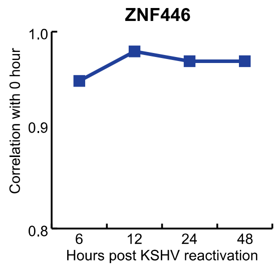

B

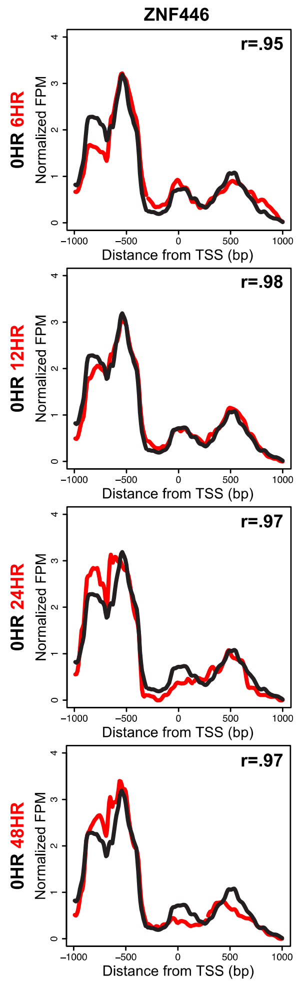

Supp. Fig. 1. (A) The correlation values between 0 hours and the reactivated state's timepoints nucleosome distribution for ZNF446. (B) The nucleosome distributions of the latent (black line) and reactivated KSHV time points (red lines) for ZNF446. The x-axis represents genomic position showing two kb centered on a TSS. The y-axis is the normalized reads per million.

A

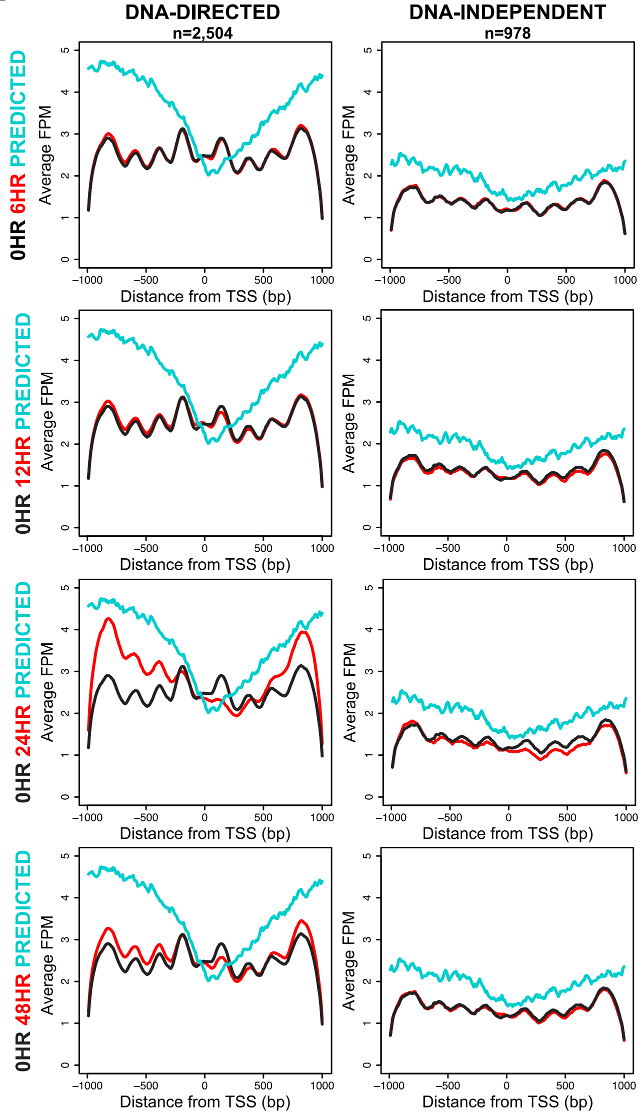

Supp. Fig. 2. Average plots for the 2kb surrounding the TSSs of the genes identified as DNA-directed (n=2,504) and DNA-independent (n=978) for 0 hour (black line), reactivated timepoints (red lines), and predicted (cyan line). The y-axis is the average midpoints per million, and the x-axis is the 2kb surrounding the TSSs of all genes classified as DNA-directed and DNA-independent.

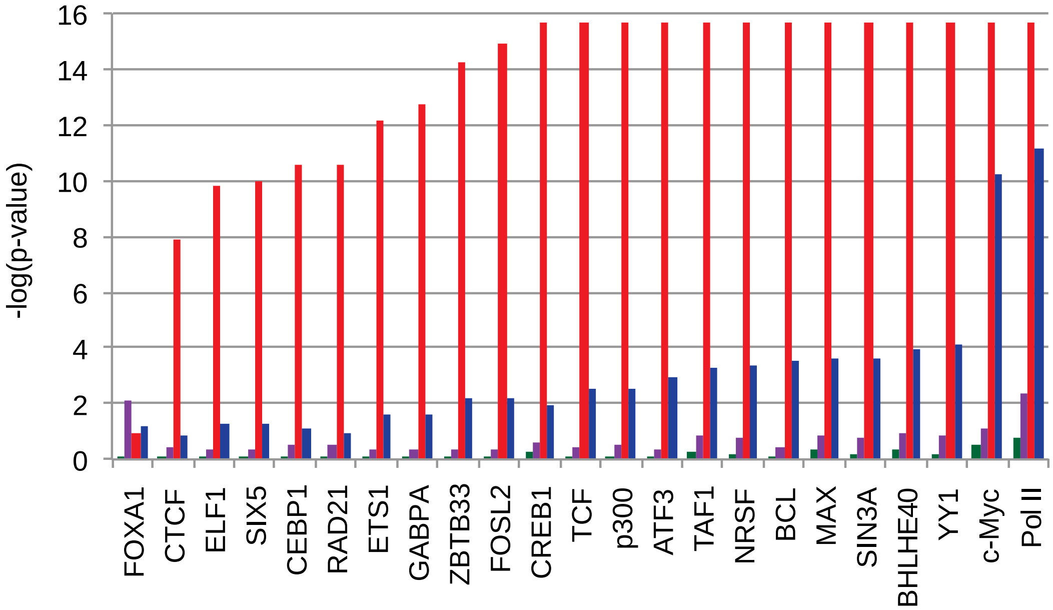

Supp. Fig. 3. -log of the p-values for the subnucleosomal fragments at A549 regulatory factor binding sites (2011) for 6, 12, 24, and 48 hour timepoints (green, purple, red, and blue respectively) compared to 0 hours.

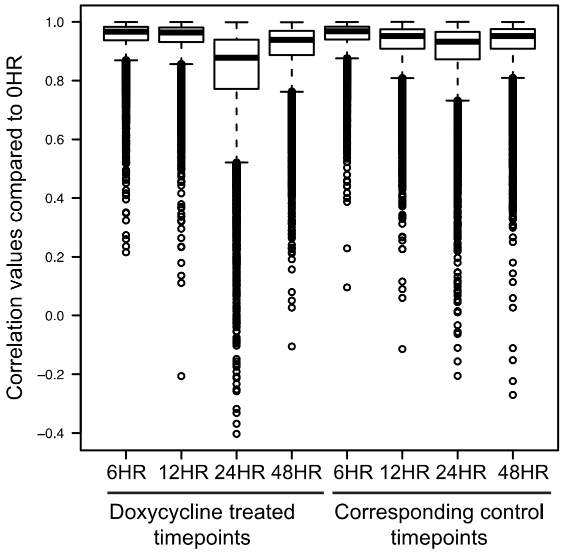

Supp. Fig. 4. Boxplot of correlation values for each TSS between iSLK.219 0 hour and iSLK.219 cell line 6, 12, 24, an 48 hours post doxycycline treatment and control iSLK .219 cell line corresponding timepoints. The y-axis is correlation values of the sample with iSLK.219 0 hour.

Supp. Table 1

Supp. Table 1. Loci classified with changes in nucleosome distributions, DNA-directed, and DNA-independent at 24 hour timepoint.

LOCI WITH NUCLEOSOME REDISTRIBUTIONS AT 24 HR TIMEPOINT	
**DNA-Directed loci**	**DNA-independent loci**
PUS10	NDUFA7
CSTF3	EXO5
ZKSCAN3	MAGEA1
KRTAP19-3	CCDC176
MRPL33	B3GNTL1
OR56B4	FAM205A
AP2B1	AKR7L
KRT10	NID2
MITD1	P2RY12
CSTF3-AS1	KIAA1984
VTA1	LINC00922
MRPL30	FAM46D
NUP35	SNORD116-16
THADA	BANF2
OR14J1	PLAT
OR5L2	POSTN
ITPK1-AS1	LAIR2
PCDHB7	CHRM2
CDKL3	MIR376A1
DGUOK	LDLRAD3
IL22	CHAD
CHMP2B	HSD17B13
MIR520C	ADIG
SPC25	C9orf57
EDC3	GBP7
CCDC172	ZFYVE16
OR56A1	LOC388948
BEND3P3	GRID2
MIR3194	GABPB1
OR5L1	FLJ42627
HELB	MIR2392

TTC14	MCM2
CLDN10-AS1	CCR5
APTX	WTAPP1
LCE4A	SHPRH
PEX13	ARHGAP18
MAGED2	SRD5A3
MAGEB3	TNFRSF11B
ZBTB41	DEFB105A
TCEANC	LOC728819
LINC00189	SNORD115-2
OXNAD1	HCAR1
SPIN2B	CYP2C8
ZUFSP	LGALS9B
VTRNA1-1	LOC100506343
GLIS3-AS1	RXFP1
LOC100288198	MRPL20
CLEC4D	CASP8
NUDT15	GPR50
LOC100133957	ITLN1
ERLIN2	IFT122
COLQ	HPYR1
PTTG3P	EVI2A
LINC00242	URB2
SNORD116-29	ACE2
FAM170B-AS1	REC8
SGOL2	LHX8
SMARCAL1	HIST1H2BO
FAM71C	SLC44A5
OR4K1	SMAD6
KRTAP13-2	CASR
OR2AK2	PRAC
ZNF268	LHFPL1
MRPL17	ABCB5
MRPS36	MAPT-IT1
C9orf3	CFI
BMP10	GAD2
GLIPR1L1	HCG17
UXT	LOC388849
OR51M1	BHLHE41
TMEM67	OR6C6
ZNF138	CYP19A1

LOC100616530	GLP2R
OR10T2	SNORD115-14
POLR3A	LOC154449
ANKRD49	MIR1203
TMEM230	HIST1H2AM
LOC151171	C11orf80
OR5AR1	CLSPN
CDK15	PKIB
C18orf54	MIR5705
OR2M4	SRMS
ETS1	ZNF607
RBM12B-AS1	SLC24A2
DBIL5P2	SLC35G5
OR10K1	LOC100505835
CCT4	MIR1184-3
TTK	SSR4P1
ZNF784	SRXN1
GPR52	GEMIN7
KRTAP19-5	MIR767
CLVS1	MIR380
CA5BP1	PSKH2
SPZ1	MAPT-AS1
OR52N4	IL1RN
SNORD50B	CARD6
LINC00888	MBD4
MRPL44	MIR3909
TMPRSS3	ABLIM3
MRE11A	RCAN2
MMP13	SNORA59B
SLC7A11	LINC00442
MIR548H3	TTC1
RWDD1	GREB1
KRTAP1-1	MIR3200
MIR4465	RPL23AP82
VPS41	CNR2
KIF18A	RNU6-28P
ADH7	LOC401134
OR8H3	SLAMF9
PCDHB5	MIR4524A
UTP6	MIR19B2
CXorf61	TUBGCP5

LAMA4	NBEA
DHRS7B	HAR1A
MIR5581	SEC14L6
SYNPO2	MIR4524B
CYSLTR2	ASPDH
FILIP1L	CLEC4A
NDUFAF4P1	SHC2
LINC00467	ASPRV1
ZNF780A	TBC1D3P5
MIR4795	PLAC8L1
OAS1	DDX25
RNF20	GLIS2
KRTAP7-1	PWP2
YY2	OBP2B
SPTLC1	FOXF1
MORF4L2	MIR503
CEPT1	ATP2B3
MIR3157	ADAM1A
ZBTB6	TAS2R16
GDAP1	PHB
MIR4781	NAGA
DEFB126	DBR1
HMGN3	SULT1A2
TMEM232	SCN11A
PNPLA8	KRTAP1-4
ZNF431	ST18
MORC2	PRL
FAM124B	EGFLAM-AS4
YIPF6	KLK9
ALG10	PPP1R1B
C5orf51	TRIM56
HIST4H4	SLC26A5
LINC00636	PCYT1B
IPO4	MIR1178
SULF1	TWIST1
FAM127C	PIN4P1
MED23	LOC100506422
CSTF2	GK2
ADAT1	SCARNA3
SULT6B1	FIS1
ACY3	LOC100506233

METTL2B	LOC100505768
METTL15	LINC00898
TMA16	PLIN5
OR13C3	IL20
WDR70	GGT8P
DPH3	UBL3
MIR4633	NAPSA
RANBP6	SERPINF2
SLC22A24	HIF1AN
OR8A1	TMEM194B
KLB	SNORD115-24
ZSCAN5A	MIR300
SLC7A9	FAM83G
HIST1H1D	C4orf26
HARS2	CISH
NDUFS4	SLX4IP
MIR4255	ING2
SNORD116-26	APOL2
OR5P2	AADAC
OR2G3	MIR507
MTFR2	GPT
LYZL2	ZNF445
ANXA2P3	TRAK1
ZSCAN12	SLC13A2
CCDC132	ZNF610
LUM	RNU6-76P
TXNRD3NB	ABCA6
FANCD2	MIR125B2
HARS	MT1F
LOC401320	MIR487B
ABCC5-AS1	CLEC11A
LDHA	LOC157273
PAK1IP1	WDR64
CCR8	OR2H1
GSTTP1	ESRRG
DNAJC2	MIR502
SFI1	MIR501
LINC00202-1	LOC100506124
BASP1P1	RPS28
LINC00864	MIR1264
MMP1	CA14

KLHL41	PDPR
ART3	TMEM52B
INTU	LOC100506123
MYBPC1	SLC32A1
PEX11A	MUC5B
NDUFA5	MIR363
C12orf4	GZMM
TSNAX	ZNF497
OR8H2	FLJ23867
LEMD3	SNORD116-2
MC2R	C16orf98
ST6GALNAC5	OR2A7
KRTAP21-2	ABHD4
CDH12	SNORD88C
LRP2BP	DDO
HCCAT5	HIST1H2BM
LIPT1	MRPL3
WDR93	TMEM174
SEC24C	CHST5
LOC100130298	GPR84
KIFAP3	PADI6
PAPD4	CA10
PYROXD1	SNORA29
IARS	MARCKS
ANKRD30BP2	MGC27345
METTL2A	SH2B2
KRTAP6-3	SNORD115-35
NUP43	MIR5688
PRPS1L1	ZNF733P
UNC13C	KIAA1211
BTBD8	ZNF534
GTPBP8	MAP3K14
IFT74	SMCR7L
GPR15	SNORD114-12
DNAJB13	GPR148
ZNF780B	ASB2
OR4L1	C8orf4
RAB3GAP2	GBP4
DYNC2H1	ACTRT2
COX15	CDRT15
KIAA0101	TOPAZ1

RBM41	OR51G1
RPL39L	LOC400940
CCDC181	USP6
ZNF92	RACGAP1P
ZNF714	ARHGDIB
ENTPD1-AS1	TIMD4
GFM1	VSTM2A
SATB1	ELFN2
LLPH	HAGHL
MNAT1	ZNF540
ELP3	TFPI
LOC145474	SNORA70B
MIR1284	MIR561
TRIM55	TCP10L2
NALCN-AS1	MIR1324
ANO4	RAB3C
FAM172A	C1QC
SNORD115-1	CLCA4
CLDN16	SCG5
ARMCX1	MIR1245A
REG1A	MIR5685
TCEANC2	S100A7A
BET1	IMPG2
PTTG1	LOC643441
OR1D2	MIR1245B
RAB5A	IFIT1B
SLC30A6	C21orf15
POLR3B	TMPRSS11GP
DLGAP5	SECISBP2
PHOSPHO2	LAX1
MYNN	NOP56
ALG10B	LOC100507362
CCDC173	LINC00838
YAE1D1	SNORD115-44
HIST2H3D	CLECL1
SLAMF7	SLC22A31
MMRN1	MIR944
DSPP	MIR4746
SNRPD2P2	SCGN
HIST1H2AA	ADCY10
MIR892B	DEFT1P

OR52K1	DYRK4
TAS2R7	MIR604
BANK1	TENM1
OR56A5	LOC391322
N6AMT1	C7orf61
LSM5	LOC200772
OXCT1	EOGT
AGBL3	CENPQ
ZNF608	CCDC135
CLUL1	C15orf62
HLA-DQB1	MUC6
SIPA1L1	MUT
IL18R1	FKBP1A
DEFB132	MIR1260A
PCDHB11	MMP11
DZANK1	SCN7A
HTATSF1P2	MYBL2
PCDHB18	CACNA1A
H2AFJ	GAL3ST1
THNSL2	CDSN
ANKRD22	SLC6A14
SZRD1	MIR4423
TRMT10A	SMIM4
COA1	MIR626
RBM7	TRIM5
RNASEL	COX6B1
MIR548AU	BEAN1
ZHX3	R3HDML
TSGA10	SNORD45A
SMYD1	FAM175A
ASB18	LINC00523
OR4N4	SNORA20
DPY30	FLJ10038
LOC286186	NOG
C2orf44	FAM81B
LRIF1	ZNF736
EMCN-IT3	MIR487A
NSUN6	KRTAP12-4
TAAR5	NCF1C
DDX58	CD79B
MIR3913-2	SUMO3

IGSF10	FGB
POLA1	TDRP
MTTP	ERCC6L2
KRTAP26-1	SNORD53
ZNF573	SNORD115-9
CD247	NT5DC2
TRDN	FLJ14107
MMS22L	MGARP
MPZL3	LINC00330
OR4N2	SNORA6
ZNF23	IFNK
ZMPSTE24	MOB2
MIR423	MIRLET7A3
EIF2S3	LOC283177
PTER	SLC5A1
TTR	MIR1238
ALOXE3	TMEM132B
OR10X1	LOC100144595
ZNF85	SPINK4
SMLR1	HELT
LARP4	MUC12
OR5A1	PCK1
CRB1	FAM90A2P
OR12D2	SNORD113-9
TCEAL8	TMEM176A
OR4C12	PRDM14
FAM5C	MIR4480
TMEM71	TCTN2
OR5AS1	COL18A1
FAM186B	MIR192
FAM229B	SYNJ2BP-COX16
OR52J3	FMO9P
SNORD89	MIR92A2
FNDC7	NEDD4
UMPS	FLJ44511
COMMD9	MTFR1
HMSD	TSHB
LINC00264	TAS2R50
RPL26	MIR548I4
ABCA12	MIR4663
MIR659	C2orf48

EID1	BVES
PEX2	RASD1
PDCD10	LOC401321
ZNF45	OR2A4
MIR4644	ITIH3
CCT8	NXPH1
LOC100506035	LOC100288255
MIR3184	CHGB
METTL25	CRISPLD2
KIAA1731	UGT2B10
THAP6	AIPL1
RNASEH2B	CCL13
ARSJ	LOC648691
TUBE1	TXK
SNAR-I	MIR4283-1
CCDC59	BTBD19
OR2J3	TMEM52
VTRNA1-3	SPINK9
KCNT2	MIR328
NOP16	UGT2B28
OR10R2	ZNF705B
RAB11A	HTR4
IRAK1BP1	C11orf94
DCAF13	SNORD116-5
EXOSC8	SNORD127
PARP11	MIR4294
OR4E2	OR2A5
HBB	EVL
SSX8	NPR3
SNORD116-25	FASTKD1
SMEK3P	PTGDS
MIR5702	SNORD115-17
CSRNP3	FAM179A
C15orf43	C10orf54
OR2T10	SNORD115-8
BNIP1	MIR4284
OR52I1	MIR3196
GHRL	ZNF83
RCHY1	TFF2
FAT3	ZNF556
ARL6	SUSD2

GIMAP4	ZNF688
SLC4A10	HAND2
UTP14A	LOC100128176
KRTAP8-1	ICAM5
MIR184	HR
OR4N3P	SNORD115-6
SREK1IP1	SGK223
THEM4	TIGIT
TRAFD1	ANAPC11
KRTAP13-3	TMEM176B
BRIP1	MMP23B
ZKSCAN8	CABP2
KDM5C	RASSF2
METTL14	PRDM16
TRIQK	LOC100128770
TPTE2P6	USP50
ACTBL2	SH3TC1
PPEF2	SEZ6L
SRP19	TMEM86B
C7orf71	CDRT15P1
LOC100270804	SYT17
MIR3913-1	DUOXA1
HIGD2A	LOC100131825
GNL2	CTSO
GPM6B	NCF2
KRTAP6-1	PRAME
ACTRT1	PARD6G-AS1
CAPNS2	NLGN2
TGOLN2	TTI1
COL5A2	LOC100505478
ZNF486	TP53AIP1
HIST1H3I	MIR3074
OR2L13	CPXCR1
RNF111	TEX21P
SRSF11	MIR378E
RPA4	CPLX1
TMPRSS11F	SNORD10
CABS1	MBD3L1
OSCAR	SNORD116-8
PTHLH	TRPV5
MUC15	RIPPLY1

SDHD	DNAH12
LYVE1	MIDN
TRIM26	HTRA4
OCIAD1	MLC1
CD4	NR5A2
WDR36	SNORD115-15
ZNF541	CPT1B
MIR215	CYBA
ZNF546	SNORD35B
CAT	BTBD6
FYCO1	KIF4A
C15orf37	SFTA2
SEC23B	GDPD3
FAM114A2	PAX5
THSD4	ZNF415
IFNA6	SGCA
SLC25A19	ANKRD24
LRRC40	DEFB1
RFTN2	TPSD1
MIR197	KCNAB2
OR9G9	RPP25
CENPW	H19
CDH26	MIR5003
OR4M2	OR2G6
TNFSF13B	C5orf38
DCAF4L1	C9orf89
CHCHD5	MIR424
GPR111	SRRM5
OR10A5	OLIG3
MIR32	MIR196A1
RACGAP1	SNORD113-7
MIR4473	MIR4739
NXF5	SLC7A5P1
OR1J4	ADRA1A
ERAL1	MIR1-1
BBS10	FGL1
KRT84	ZNF625
KCNAB1-AS2	ZNF735
MIR4650-2	SNORD110
MFAP3	GOLGA2P5
TMEM18	RDH12

SKA1	FASN
RPL5	MIR4289
MIR933	TAS1R3
RPSA	EDDM3B
OR2A25	SNORD19
LINC00616	FAM212B
TIMM8B	LINC00398
MMP26	LINC00899
LRG1	SULT4A1
TNN	ARHGAP35
AVPR1A	DLX6
IQCF3	SNORD116-13
KRTAP23-1	PIAS4
ZSWIM2	MIR514B
FXR1	IL2RG
POU6F2-AS1	CALCA
POLR1B	LRRN4CL
LOC100128993	FUT7
ARHGAP15	GALNT8
ZNF562	CYP2D7P1
SPOCD1	SOX3
HAVCR1P1	CHKB-AS1
ILVBL	LOC100129083
LYZL1	TMEM240
EFCAB3	KRT14
AGBL2	RGS8
OR5K4	AUTS2
KRTAP13-1	SNORD115-39
MIR1913	OAF
ZRANB1	MBL2
TRIM69	MIR656
POTEE	LOC100129055
SNORA13	SEMA3G
EBLN2	PAQR5
ESF1	HOXB-AS1
STX7	TACC1
KPNA2	ZAP70
TPI1P3	ABCD1
BTNL8	FAM181A-AS1
TRMT5	DUOX1
POLR3F	DEFB106A

CWC27	PDE10A
RGS12	MKNK2
NDUFAF5	MIR4690
TIMELESS	SNORD115-17
NUFIP2	MIR591
CLDN25	GSTM5
HRNR	OR5C1
LUZP6	TIMP1
LINC00113	TCFL5
FAM83A-AS1	DEFB106A
TMC3	MIR876
ZNF155	FAM171A2
OR4D11	MIR4535
PKD1L3	DEFB107A
MIR3151	GPR153
MIR551B	SLC13A5
LOC150568	FBXL16
CHAC2	UBE2QL1
ST5	MIR651
SLCO1A2	SPATA31D5P
C2orf42	KLHDC7B
RNLS	LOC283332
LOC100507244	ZNF813
SPATA6L	PSG4
CCNB3	MIR208A
MAP3K19	C1orf140
KRTAP4-12	MIR4706
KRT39	SERPINA13P
C2orf49	HOXD3
ZNF33B	LOC100132078
CYP2G1P	TAF6L
MIR4263	MIR1236
OR10A4	TUSC7
MIR3666	MIR4646
C1orf116	CEND1
LOC154872	MIR4292
AICDA	CLEC12A
ADH1C	E2F7
SERPINB8	SNORD115-22
EXTL2	CHKB
LOC727982	SOHLH1

GTPBP10	TMEM257
FAM106CP	LINC00112
RBM34	PUSL1
OR4F6	IL19
OR5B2	RFPL1
TCEAL3	LOC100507300
MPHOSPH10	MIR589
FAM227B	VRTN
LOC100506844	CLCNKA
MMP7	DGKK
CSRP2BP	MIR4684
SOGA3	POTEM
IL9	A1BG
SCML4	OR5AN1
PPIG	CAV3
ASH1L-AS1	FAM133B
TOR1AIP2	ALDH1L1-AS1
TOR1AIP2	ABHD6
OR4K13	SNORD115-25
PCNAP1	GALP
SCFD2	MIR431
HIST1H4K	OR11H1
DSG3	LIME1
N4BP2L2-IT2	FOXN3-AS2
ZCCHC4	LINC00163
IL15	FZD10-AS1
LINC00570	LOC151484
CDKN3	BFAR
SDF2	PEX10
MCEE	SERPINA11
LOC340515	SNORD115-31
MRPS15	LINC00951
TECTB	MIR324
SERP1	OR2AP1
TGFBR2	CD1E
PTGS2	MIR432
OR1Q1	TRIP6
NSUN3	LINC00890
RAD9B	TBC1D9B
ZNF620	TMPRSS4-AS1
LOC81691	SNORA51

ASAH2	LOC400620
LOC100506025	P2RX1
ZNF718	ASS1
MIR490	MIR4763
ADAM20	PPDPF
SNRNP27	NCDN
A4GNT	ZNF284
MLF1	MIR33B
PRPF38B	GPC3
TMED11P	CALHM1
TAGAP	BDH1
FGG	ITGAL
URM1	FAM95A
PSME3	PTH
MIR3910-1	SNORD23
HSD11B1	MIR1179
ITGAD	SYNPO
RBM19	PRAMEF22
ADAM21	ADAMTSL5
GZF1	GLRA4
C11orf71	CBX6
NAA20	TMEM53
EXOSC3	SEPT5-GP1BB
LINC00929	C22orf42
SUPT6H	WISP2
FER1L6-AS1	SNORD115-10
MIR3136	MRPL1
SCYL2	DTX2P1-UPK3BP1-PMS2P11
MIR4716	SFTPD
MIR504	SPERT
STAT2	LOC339240
DSE	FAM160A2
IL17A	MIR4710
UBR2	MIR4451
CHURC1-FNTB	GNAT2
NAP1L2	PPM1A
PNO1	TEKT4
CLEC5A	SNORD116-12
RBM26-AS1	MIR939
HARBI1	PCSK2

PPP1R8	SIGLEC12
OR4K14	TLE3
DEFA10P	LGALS16
LRRIQ3	SSTR5-AS1
STAR	PRSS22
IGBP1	LOC284688
CD3E	BTN3A1
USH2A	SLFNL1
MIR4650-1	VSTM2B
VPS29	ZBTB32
OXR1	WNT2
FOXR2	IGFALS
MIR548B	SNORD115-40
SLC38A6	HOXA6
OR56B1	MIR4745
FPGT-TNNI3K	METTL12
TEPP	RPS13
TTC5	C9orf62
TSPYL1	RPRD1B
C7	LOC286083
OR8U8	HCN2
KIF2C	MYCN
ABCB1	ITM2C
OR4C11	TMTC1
MANEA	MEGF6
LOC100506085	SNORD62A
PRAMEF12	PPEF1
DIEXF	MAGED1
KIAA1429	FGFR3
PCDHB9	IZUMO4
PSMD10	OVOL1
OR6C76	NKX6-2
MIR4317	RESP18
MOCS2	FEZF1
GPX5	KCNT1
AMY2A	C1orf228
KITLG	CNKSR1
RAD51AP2	WNT6
OR51L1	TDO2
DKFZp434L192	LOC100288123
RAD51AP1	CST4

C14orf178	TREX2
LINC00493	OR8K3
HMGN4	DCAF12
ART4	MIR375
STK11IP	ACSM2B
MEIS1	C11orf34
CCT2	PRR13
OR2A2	SNORD115-11
NUDT6	PSMD5
MYADML	SPOCK1
SNORD54	MIR4313
SNORD116-3	RPUSD1
WDR52-AS1	MAMDC4
KIAA1009	LRFN4
MASP1	LOC100128593
MSL3P1	HGFAC
SDAD1	CTAG2
ATG4A	MOB3A
LOC100130849	MGC12916
DSG1	TUBB2B
KRTAP9-9	C19orf81
LCE1B	A1BG-AS1
SH3BGRL2	LOC100131094
DUSP11	U2AF1L4
SNORD116-10	TBC1D26
GORAB	PRSS57
OR52A1	HPCAL4
CETN4P	BRD1
TJP2	MIR1289-2
NCAPD2	DGCR5
NEK11	SMIM21
WRAP53	LOC100129034
HAPLN1	DEFB113
LOC286367	SATB2-AS1
ALG14	PRSS46
KRT20	CHST15
SLC7A2	SIX2
OVCH1-AS1	OSM
LINC00837	FNDC5
SNORD90	PRSS55
MGP	DNM1P35

OR52N5	LOC646736
OR10A2	MIRLET7B
KRTAP1-5	GMPR
ZNF880	MMEL1
SPATA31E1	NTNG2
NBPF22P	MAD2L2
UOX	ANO9
LCA5	RNU5F-1
LRRCC1	PTGES
OR1E1	MIR3621
BMPR1B	F8A1
MIR4720	LINC00632
OR4M1	APOL4
MIR101-1	WBSCR22
MIR323A	SNORA57
OR52R1	VIPR2
MFSD8	ASIC2
TMEM212	SLC18A3
DHX33	LOC400558
GRIA1	MDK
UBQLN3	GIPC3
OR8H1	ASPHD1
DHRS9	LYPD8
ATP13A4	BTK
COPS8	CCDC54
PNPT1	BARHL2
TMEM126A	C17orf74
ARMC2	SNORD65
ZNF271	MIR216B
MIR4727	CBS
SIAH3	FLJ36777
PRDM2	CNTN4
LOC550643	CCDC114
OR2A12	NSUN4
ADH1B	OSTCP1
NNAT	NAT16
CHRNB3	PNMA2
MMADHC	CITED1
OTUD6A	LCN10
PCM1	MICALL2
C9orf153	MFF

MNS1	CEBPA
ATG13	PGM5-AS1
OR4N5	LOC285768
RFC2	SNORA70
DUSP12	PRDM6
LOC285501	PRKAR1B
ZSCAN30	GRM3
CLDN17	CP
BTD	DUOX2
HIST1H4A	PSAPL1
OR4D5	CSNK1G2-AS1
ECD	ONECUT1
ST13	CCDC154
ASTE1	ZBTB48
C13orf35	CEBPA-AS1
NEUROD4	PLEKHD1
SUGT1	SOX8
HBD	SNORD114-29
DEFB125	SNORD115-30
F5	LOC283731
OR5F1	SOCS2-AS1
LRRC52	LCN6
DNASE2B	GJD4
SCLT1	SLFNL1-AS1
NOBOX	MOCOS
AREL1	WWC2
IGSF6	LOC100130238
CXorf56	GRIN2A
MUC7	COL19A1
PARP6	SNORA65
CT64	SLC5A9
LINC00920	PIRT
TAAR8	TAGLN2
CHD1	SNORD115-5
OR2T34	MIR511-2
LINC00889	H2AFB3
DKK2	MIR3605
MIR5579	MIR210HG
PCDHB3	MED15
LINC00207	GRIK3
OR4A16	BCORL1

OR10A7	PATE1
SPIN3	RAX
GALK2	CCDC153
CTSL1	MIR26B
MRPS9	AATK
FAM149A	FGF8
LOC284933	LOC100130899
TUBAL3	SPATA8
EQTN	FOXB2
TRAPPC13	SPATA8-AS1
TRAPPC13	MIR4656
BCO2	TGM6
YME1L1	MIR409
TRIM23	ANKRD35
HIST1H2BA	FAM57B
MIR29C	PANK4
SPANXN2	DNAJC30
PSMA1	MIR411
GNG5	CACNA1H
TAS2R8	DOK7
UBR1	LHX5
PPWD1	KLHL17
IL13RA2	DGCR11
ARPP21	CTRB2
ZSCAN16	NPAS1
AKR1C6P	C1orf233
TFF3	SNORD115-33
SAAL1	MIR3183
NTS	LRRC56
OR8G2	YWHAE
PMS2P5	RNASE11
ZNF317	TCAP
NBPF15	PIP5K1B
NUDT9	ZSWIM1
NAV3	PABPN1L
OR8I2	EGFR-AS1
MIR941-3	STUB1
PAAF1	FLI1
GARNL3	DNASE1
RIT2	FAM132A
CHMP4C	MYLPF

OR6C74	KLHL34
ZNF404	SNORA71C
FUT10	MIR412
TRAPPC6B	SIX5
LAMTOR5-AS1	ZNF141
TAF9B	MIR1185-1
TMEM92	DLGAP4
HIST1H1B	ZNF180
P2RY13	OPN1SW
OR52N2	SNORD115-4
LOC100129316	INSM1
MBL1P	ACAP1
MMP8	MDGA1
SPRY1	MIR4641
FCF1	ZNF701
KRR1	MIR4640
OR10G4	CCDC36
LIN7C	MIR4760
CATSPER1	TBKBP1
MC4R	IL21R
CYSLTR1	EPHA10
ZW10	GRAMD4
SNORA75	MYCNOS
FAM149B1	AQP7P3
HFE	CRHR1
C14orf1	THBD
C15orf52	RPS6KA4
OR52M1	DOK3
FAM13A	MIR210
EBNA1BP2	MIR3977
MIR4476	TPPP2
MIR5087	CECR3
OR2T1	GPR132
ECT2	MIR4261
TPRG1-AS2	UBE2J2
PLCB4	AMH
LOC643837	LOC100133920
TLK1	SCARNA12
LRRC69	HS6ST2-AS1
CENPK	PRSS3P2
ADAMTS9-AS2	PRSS27

LINC00672	MIR410
OR5J2	BRSK2
ZNF615	ACHE
SAMD9L	LOC440905
OR52I2	ALKBH6
SUMO1P1	PNMA3
CWC25	DPP7
HTR3A	FOXI1
CDH18	TRIM71
VSIG1	MPV17L
MMP16	IL36G
MAGEB16	MIR124-3
CCR4	RNU6-31P
CTH	MIR369
LRRC37A3	EFCAB6-AS1
TTLL5	MIR5699
ANKRD62P1-PARP4P3	LYPD2
PRB4	LOC286297
DAPL1	SNORD115-11
LINC00710	WWC2-AS2
LINC00332	FFAR1
POU5F1P4	FUOM
H3F3C	LANCL3
FAM115C	GPATCH4
PCDHB2	ZNF350
ANGPT2	LOC100169752
PRKAG1	NBR1
C21orf37	BGN
RFESD	LOC100133461
LOC253573	KIF26A
SPRR2C	KRT73
USP47	LOC100130348
BEND7	ASGR2
MIR4759	TMEM132E
MIR892A	CDCP2
PELP1	CCDC116
HES1	FCN2
XCL2	GP2
ZNF33A	NIPAL3
CYP20A1	SMOC1

EYA1	MAZ
LOC100294145	LINC00696
C9orf53	NOTUM
RGPD3	SOX11
C10orf90	MIR299
DEFA5	EHD1
CELA2B	LOC285484
OR4B1	MIR1914
SPINK7	ARHGEF16
ANKS4B	POU3F1
NMS	SEZ6L2
KRTAP3-3	FAM107A
ASB9	DMKN
HEPH	NACC2
AP1G2	FGD3
SYT4	MIR1250
HACL1	KCNN2
RMST	FZD10
STPG2	C9orf173
ATF2	TNFRSF18
ERV3-1	SLC35G3
STAG3L2	SOX21
WDR31	MIR382
KLHL4	CCL3L1
NPY1R	PPARA
RSRC1	PCP2
LRR1	TNFRSF6B
IBSP	GFPT2
MIR30E	C20orf27
INIP	MSANTD1
MIR1205	IFNL3
EXOG	VGF
CCDC86	ZCCHC2
GSTA7P	BCL6B
LOC100379224	C20orf201
STEAP1	PRR22
OR4K15	SNORD115-38
TRIM6-TRIM34	ADRA2B
LOC284100	GNG13
OR5M3	DMRTC2
ZNF107	PXDN

WBP5	SLC11A1
FER	SLC6A2
EAF2	TYMP
MIR3910-2	MIR3689A
RPA2	BATF2
PLCL2	MIR3689F
MIR335	
DIRAS3	
CRHR1-IT1	
TTC27	
TCEAL1	
SNORD109B	
MGC16142	
KLHL2	
TMEM184C	
OR5K3	
SUMO4	
SNORD114-7	
PCCA-AS1	
MBIP	
PIP5K1P1	
CLCN4	
ERI2	
TMEM59	
CCDC28A	
LOC389043	
PLCH1	
PPBPP2	
PGBD1	
PLAC1L	
ACPP	
NDUFA9	
OR51A7	
LPCAT2	
TRIM68	
LOC284661	
OR13F1	
GADD45GIP1	
FETUB	
KRTAP15-1	
PRKAB1	

CBR4	
TSPAN19	
TOMM7	
ESCO2	
DBT	
OSBPL10-AS1	
SLC7A8	
B4GALT3	
GLYAT	
WDR33	
MBNL3	
LOC401463	
NXF3	
SPANXN4	
LOC646329	
PPP2R2D	
CCDC58	
MIR138-1	
SCFD1	
NUBPL	
OR6C3	
TRAF3IP3	
MDM1	
OR10V2P	
LINC00457	
OR5AK2	
AQP4-AS1	
C2orf47	
RPL13AP3	
OR2A14	
GPR65	
HMGA1P7	
MIPOL1	
MAP2K4P1	
RPLP0P2	
PIGK	
ZNF90	
PDE12	
AADACL2	
SLC35B3	
OR52D1	

ST6GALNAC1	
CDH9	
CAPS2	
LRRIQ1	
BMX	
LOC101559451	
LOC285593	
SNRPD1	
MIR758	
FAM205B	
HLA-DPB1	
NKTR	
CCDC65	
CEACAM6	
TXNIP	
C6orf58	
PIK3CG	
ARPC5	
LINC00312	
NSRP1	
LINC00944	
IFNA8	
KRTAP6-2	
NLRP9	
OR6X1	
POLM	
SDE2	
BCKDHA	
SHFM1	
SPRR1B	
MIR3976	
ITGB3BP	
DRAM2	
FERMT1	
OTOL1	
IFNA22P	
FAM115A	
LINC00174	
TSPAN12	
CTXN3	
MIR3198-1	

AEBP2	
C2orf15	
PHACTR4	
OR4C15	
NUDCD3	
LOC644669	
LOC100505989	
PLK4	
KCNH7	
RNU6-66P	
GPR156	
RAET1K	
SPATA31D1	
LINC00347	
CCDC148-AS1	
MYH1	
OR6M1	
IQCF5	
MIR4464	
LINC00478	
KRTAP19-2	
HMP19	
PCDHB14	
ZNF100	
EXOC4	
WBP4	
CNBD1	
FANCL	
HSPA8	
LOC100505549	
ZNF257	
NOX5	
OR5R1	
LOC100289673	
LIPN	
MIR542	
CNTF	
ARMCX3	
OTUD6B	
LOC100131067	
LOC644649	

OR2L5	
BDNF-AS	
TRIM21	
GZMK	
KCNE4	
C1D	
MARK2	
SPATA31C2	
LOC550112	
IYD	
MIR4306	
HCG23	
RHOXF2B	
PMFBP1	
MYSM1	
SKIV2L	
ALKBH3	
EDAR	
TECTA	
ZNF432	
ARL6IP5	
MYO16	
CPO	
GPR183	
LOC400654	
ADAMTSL1	
TMEM45A	
LOC100507066	
KRTAP4-11	
IQCB1	
SLC25A21	
OR2B3	
GCM1	
ZNF589	
ACMSD	
LRRTM4	
FANCG	
KPRP	
PRSS16	
RPL21	
HSD52	

HIST1H3H	
LINC00559	
LINC00865	
LINC00115	
SS18L2	
LOC650226	
EMP3	
NCKAP1L	
SPRR2E	
GINS1	
MIR4639	
OR51B6	
HBBP1	
RSU1P2	
VTCN1	
WFDC8	
DRG1	
OR5B17	
ALDH1B1	
MSS51	
MIR873	
PIGB	
RNF144B	
MRPL22	
LRRC18	
MIR206	
CNTNAP4	
KRT77	
MIR4643	
FAM47B	
LINC00348	
OXSM	
PTGFR	
MIR4295	
ASPN	
SNORD66	
C14orf23	
UPP2	
BORA	
MIR4327	
KLRF2	

MTRNR2L4	
TAT	
ATAD3C	
GABRA1	
GCA	
BUB1B	
C11orf82	
MIR3671	
TENM2	
LOC100287010	
NEIL3	
PCDH18	
HHLA3	
ZCCHC9	
CDKL2	
PCBP3	
GAL3ST4	
SPRR2A	
TSACC	
SNORA79	
SNW1	
GCNT1	
CAPN14	
LOC100507462	
CNGA1	
CDKAL1	
ZNF121	
OTOR	
FKSG29	
EFCAB7	
C12orf40	
CAV1	
NDST4	
PPP1R1C	
MKRN3	
CADM2-AS2	
LOC550113	
ANKRD13C	
CTLA4	
TXNDC9	
ZNF536	

PAK3	
SNORA70F	
SPRR2D	
ASB15	
ANGPTL1	
DOPEY2	
DOPEY1	
AQP9	
ZCWPW2	
MIR5582	
LOC652276	
HUS1	
TBC1D19	
OR2L3	
ATP8B4	
EXOSC9	
SLC39A12	
KBTBD3	
GTF2A2	
SMPX	
NDUFA4	
DISC2	
COX7C	
SMTNL1	
UBAP1L	
MAP2	
TJAP1	
OR5M11	
MYH4	
MIR5681B	
RNF146	
CCDC7	
OR4A5	
OR51T1	
C6orf7	
LINC00379	
ZRANB2-AS2	
SLC9B2	
LAMTOR5	
SMIM9	
MARK2P9	

LRP10	
DFFA	
RBKS	
CLK2P	
IL36RN	
EPN1	
UBC	
ZC2HC1B	
PNISR	
MIR4804	
CENPP	
DEFB110	
BCAP29	
MIR5681A	
GRIK2	
OR1D5	
NLRP8	
IFITM4P	
ALDOB	
NOSTRIN	
MIR3622A	
LINC00359	
LINC00160	
MIR4272	
NARS	
LINC00343	
CXCR2	
CD1B	
EIF3H	
ARHGAP25	
GYS2	
ALG13	
EIF2A	
MIR3134	
MIR320C1	
TSPAN13	
CACNA2D3-AS1	
UGT8	
EIF2B2	
MIR3921	
ZNF225	

DDX18	
SCARNA5	
TYW5	
GTSF1L	
C8B	
SNORA41	
SUCLA2	
C22orf31	
CETN2	
MT1X	
VSIG4	
RABL3	
RNU6-34P	
TPD52L1	
IFNL2	
LOC100288814	
KRT222	
LOC100506207	
QRSL1	
MIR181B2	
C6orf52	
RBM26	
PNLIPRP1	
BLOC1S1	
DHDDS	
RTN4IP1	
IL21	
RNPC3	
EMC1	
LINC00670	
KRTAP10-6	
SZT2	
RSL1D1	
SPACA7	
NPSR1-AS1	
CSTL1	
RUNX1-IT1	
MOB3B	
MIR1911	
MIR3653	
BCAS3	

SNORA40	
C6orf163	
SNORD2	
SLC15A2	
CD74	
KRTAP20-2	
OR4A47	
TFAM	
FLJ33581	
PRDX6	
SPANXN3	
VWC2L	
LOC100128531	
ATP8A1	
TEDDM1	
WNT8B	
RGS3	
RIMBP2	
TBC1D21	
EHF	
PDCD6IP	
TIMM8A	
HOXB3	
FSIP1	
SULT2A1	
VNN1	
SEL1L2	
C3orf14	
TGM4	
ANP32C	
MPZL2	
IFNA16	
KCNQ5-AS1	
IER3	
ZNF311	
SNORD32B	
LOC100505495	
SMC2	
LIPI	
OR5I1	
LRGUK	

CDHR3	
KRTAP9-4	
HIGD1C	
OR14I1	
OR5B12	
CLEC1A	
OR2AG1	
MURC	
NRXN3	
MRTO4	
SNORD114-6	
KIAA1644	
MTPAP	
MRPL42P5	
C5	
KIAA1024L	
CCDC155	
FABP1	
C6orf118	
OR10J1	
COX10	
MIR889	
GARS	
RPS26P11	
CALCRL	
CASP12	
ORC3	
NKIRAS2	
MRPL47	
GABRG1	
MYH13	
XPNPEP3	
ZNF32-AS2	
SNORD116-19	
SNORD115-11	
OR10G8	
CCL8	
MIR196A2	
ZNF208	
LOC149373	
SCN2B	

KRTAP5-11	
ZRANB2	
TAF9	
TAF9	
CYP2B6	
LOC653712	
MBOAT4	
MIR5089	
LDLRAD4-AS1	
AQP8	
PACRG-AS1	
EIF1B-AS1	
OR9Q2	
MIR613	
OR5E1P	
CYP4A22	
TNIP3	
PYDC2	
TAAR9	
DNAH14	
TAS2R10	
LINC00626	
CTSL3P	
HTR2A	
OR8J3	
NUGGC	
SPATA5	
KIRREL3	
SPATA12	
GYPA	
SLC8A1	
C10orf12	
C16orf97	
SNAPC1	
IFNA7	
LOC100129138	
NOL8	
MIR4743	
FER1L6	
SLITRK1	
SERPINB7	

LINC00615	
ATF7IP	
PRKG2	
ZNF774	
C17orf112	
TMEM167A	
SIGLEC15	
MIR1200	
CASP9	
C12orf66	
ATAD1	
SLC17A6	
LOC339568	
MIR29A	
DYNLT3	
STYK1	
ENTPD1	
ATP5J2-PTCD1	
XRCC4	
GPR22	
MIR4474	
VBP1	
MYH3	
EIF1B	
OR51E2	
MTF2	
PNMA1	
NAGK	
SERPINA6	
FEM1C	
RD3	
GPHB5	
MST1	
UBA6	
OR2D3	
LOC642361	
LCE3E	
MIR4471	
MSH4	
PPAPDC1B	
ANP32AP1	

LOC339505	
C11orf49	
SEMG2	
LRRC10	
PNLIP	
LRRC17	
SETD4	
SUPT3H	
MGAT4C	
KLHL28	
TBC1D23	
ZNF738	
LOC100128361	
C14orf105	
RRN3P3	
ZNF597	
LOC151475	
MED8	
BBS1	
MAP1B	
PCDHB17	
POLE4	
ZNF300	
MIR592	
LOC100500773	
LOC100129213	
HCG18	
HSPB3	
CD2	
NLGN4X	
MLKL	
SNORD67	
OR51G2	
TREM1	
SNORD20	
SNHG3	
CALD1	
OR13G1	
MIR4417	
FANCF	
MIR3938	

SNORD116-18	
NAALADL2	
SHROOM3	
TENM3	
OR2D2	
MIR4446	
METTL21EP	
NR1H4	
CAP1	
LINC00423	
RGS13	
WFDC11	
CYMP	
COX10-AS1	
ZNF214	
ADH1A	
UBE2U	
OR7E24	
OR5D14	
ERCC6L	
PITX2	
SMAD5-AS1	
RBBP9	
LOC1720	
NF1P2	
ARMCX6	
ESD	
CXCL6	
PAX9	
SNORA74A	
DNAJC27	
UBA1	
CALCR	
DNAJC5B	
WDR5B	
SLC15A5	
CLDN8	
CSN1S2BP	
LILRB2	
FLJ45974	
PRRC1	

OR5D16	
AZU1	
OR2Z1	
OR2G2	
MED11	
PRRG1	
LOC100506497	
LOC286442	
NDUFB5	
SLC6A15	
SCRG1	
MIR3129	
NDNF	
LOC283440	
LINC00381	
MIR340	
NLRP11	
WDR65	
CEP44	
RPL24	
HTR3B	
ATP5EP2	
PABPC1P2	
MIR1912	
MAPKAPK5-AS1	
TLR1	
LOC100507632	
RARS2	
TPRG1	
MIRLET7G	
MIR181A1	
DCLK3	
FCRL3	
ZFP57	
LENEP	
NLRP4	
SLC41A2	
RBMX2	
HIST1H3C	
BTN2A1	
MIR4536-1	

ZNF770	
KPNA5	
PGK2	
GDPD1	
ZNF407	
LCE6A	
LOC100130451	
HIST1H2BN	
TMPRSS11BNL	
POGZ	
ELAVL4	
NELL2	
CLEC3A	
CLCA3P	
PRAMEF8	
IFI44L	
MIR3622B	
SPATA9	
OR8D1	
PCDHA12	
SCARNA7	
PFKFB1	
STAP1	
SNORA50	
CHORDC1	
MNDA	
RNF7	
GPRC5A	
ARHGEF33	
ORC5	
MIR4288	
ARHGAP31	
TTC3P1	
C12orf74	
RBL1	
PLS1	
DNAJC27-AS1	
FOXRED1	
DCAF13P3	
OR6C65	
SUCLG1	

TNFSF4	
TMEM141	
C16orf3	
TMED10	
CDRT15P2	
WDR61	
DBI	
FCAR	
NF1P2	
OR8S1	
OR5H6	
LOC339788	
SFTA1P	
SLC17A8	
PCDH9-AS3	
IFNA5	
ITPKB	
HYAL1	
LMOD2	
COX7A2	
MIR5586	
STYX	
AWAT2	
HK2	
MARS2	
SAMSN1	
B3GALT2	
FABP6	
LINC00883	
DIAPH3	
MIR3646	
CFLAR	
GPR18	
MAGI1-AS1	
UNC93A	
ANP32D	
SAR1A	
LINC00208	
WDR17	
LOC100129345	
SPINT4	

MIR500B	
MIR1253	
ZNF207	
OR2L1P	
DNAH3	
POLR2K	
DDX3X	
TXNDC8	
DBF4B	
SPIN2A	
ARRDC3-AS1	
TIE1	
OR5A2	
GNAS	
MIR488	
BCL2A1	
MIR3145	
POTEB	
C6orf70	
MZT1	
UBASH3A	
MIR4434	
DLGAP1-AS5	
DDX27	
KLHL12	
PRKACB	
PIK3CB	
SERTAD4-AS1	
DLD	
OMA1	
MIR486	
GABRA6	
ALDH8A1	
FABP9	
TPH1	
EXOSC5	
PBOV1	
FOXP2	
GOLGA6L7P	
COL8A1	
CD1C	

OR10W1	
ZNF675	
FCRL2	
OR52E6	
SNORD116-1	
LIPJ	
CCBP2	
ACSM4	
RERGL	
LOC100289511	
RPS15AP10	
EBLN1	
MIR99A	
DPM3	
MIR23B	
RPAP1	
ACN9	
NAIP	
CFB	
LINC00851	
TAF1B	
MKRN9P	
MIR4319	
LNX1	
SPATA19	
CYP3A7-CYP3AP1	
LOC150935	
TAS2R60	
MMP12	
ADAM7	
SNORD114-23	
IQCF2	
BLNK	
MIR653	
MIR3152	
CCRL1	
PPIP5K2	
DLEU7-AS1	
SLC14A1	
KRTAP5-4	
PRPS2	

FAN1	
VN1R5	
CD19	
PLA2G2F	
OR4C16	
MIR128-2	
RASSF9	
SPAM1	
NYAP2	
ATXN7L3B	
DKFZP434K028	
ZNF711	
NDUFAF1	
KLHL9	
ENPP3	
SUPT20HL1	
ZNF280B	
IMPG1	
KIAA1107	
PLA2G12A	
GAP43	
LOC100506834	
CHMP1B	
MYT1L	
SNORD115-11	
OSTN	
HADH	
PTP4A1	
MED4-AS1	
CCDC140	
ZBBX	
AURKAPS1	
TRPC7	
GPR113	
DHX32	
MIR221	
RNASEK-C17orf49	
FAM65C	
PATE4	
ASB17	
AMPD2	

NOX4	
RHOH	
CD5	
MIR1279	
PHEX-AS1	
MIR548O2	
ZNF32-AS1	
ABCA13	
TFB1M	
COLEC10	
MIR1197	
CLLU1	
INE1	
LOC91450	
MIR627	
MIR384	
SNORD115-27	
PLEKHA5	
AGTR1	
LOC285889	
KRT1	
CPA6	
CEACAM3	
C10orf129	
MIR4500	
LGALS8	
DDX20	
BDKRB1	
CYP4A11	
MIR4531	
OR8G1	
TXLNB	
PRB3	
SOSTDC1	
OR7D4	
ZNF850	
CNOT2	
ANXA9	
CLIC2	
KRTAP19-8	
CLDN9	

TCTE3	
ACYP1	
C17orf105	
CCDC74B-AS1	
FCGR2A	
LINC00488	
MIR105-2	
LINC00907	
SMARCAD1	
DAAM1	
TNFRSF10A	
SAMD12-AS1	
IL24	
MTUS2	
SRPR	
C7orf72	
NPS	
CTSL1P8	
MIR448	
LOC100507577	
KIAA0087	
NBAS	
MIR4744	
LOC146481	
SNORD114-28	
SNORD51	
LINC00323	
NRG4	
LINC00476	
MIR4529	
LOC340074	
MIR3162	
SPICE1	
CRBN	
ANGPTL7	
ZNF676	
MIR5188	
LCN15	
PAR1	
OR51A4	
SNORD9	

TPK1	
SUGT1P1	
OR4D6	
SKOR1	
F9	
SP140L	
SNORA26	
GPATCH3	
MUSK	
RPL23P8	
NOC3L	
GLTSCR1L	
FAM26D	
LINC00238	
SCOC	
CEACAM21	
MIR764	
VPS37A	
MIR4778	
MIR676	
ADAM20P1	
SPATA7	
TIAM2	
LINC00474	
LINC00548	
SLC37A3	
LRRC37A11P	
NEMF	
MIR3924	
C7orf76	
MIR181B1	
TUBB1	
PHYHD1	
LRRC19	
PSMB1	
POTEF	
AQP4	
KRTAP12-3	
LINC00857	
SNORD115-28	
KAAG1	

ACBD6	
LINC00460	
PRDM1	
CNOT7	
SNORD114-24	
CCRL2	
ADAM28	
WDR44	
IL5	
OCRL	
GPR119	
PICK1	
LOC494127	
TGM7	
UBB	
TBC1D28	
PPY2	
MIR581	
CCDC147	
LOC389641	
IFNA17	
SAMD7	
TKTL1	
RPUSD3	
MIR4273	
ACSM2A	
C1QTNF6	
SCARNA6	
S100B	
B3GNT5	
ZNF645	
LOC100506393	
MIR891A	
GM2A	
MIR3619	
CERKL	
GMNN	
MIR888	
MEPE	
C19orf18	
LINC00159	

RGMB-AS1	
ACVR1B	
MIR450B	
GTF2E1	
PPP1R2P9	
DLEU2L	
OR4F15	
ATP2C1	
LOC285441	
LOC285441	
TNFSF15	
LGALS1	
ARG1	
FBXW2	
MIR3978	
LOC100506136	
MIR4449	
KIR3DL3	
UQCRFS1	
AKR1B10	
RPL23AP7	
BRE	
GNL3	
SETDB1	
SNORD28	
CTAGE5	
OR2L2	
HIST1H1T	
FAM225B	
MIR600HG	
CFHR3	
COL3A1	
RAB9A	
CYP39A1	
PRSS58	
GPC5-AS1	
CYP1A2	
LOC100873065	
CSTA	
CCL22	
ALKBH4	

CCIN	
AGTR2	
GPR63	
LOC100505658	
MIR302B	
LOC100288842	
SNORD114-13	
SLC25A35	
GPR151	
PPBP	
TYK2	
NINJ2	
SCIN	
TSPAN16	
PDE1A	
MYO3B	
SNORA62	
LINC00293	
LOC100132781	
KCNU1	
SPTBN5	
COL10A1	
TMEM242	
MRPL54	
MIR30C1	
ATP1A4	
VPS39	
TMEM27	
ORAOV1	
OR5AU1	
EPHX2	
OR3A1	
SCARNA10	
GFRAL	
RNF123	
RPF1	
C19orf80	
DQX1	
PDE6A	
TMBIM6	
MYH8	

SNORD115-7	
SNORA16B	
MIR1206	
ILDR1	
RFX3	
FAM45B	
BTAF1	
SH3GLB2	
DEFB109P1	
C11orf63	
ITIH4	
TMEM44-AS1	
CXCR2P1	
SAMSN1-AS1	
DNAH17	
LINC00502	
NTRK3-AS1	
OR5T3	
CCR1	
FMO2	
ERO1LB	
EFTUD1	
CSF3R	
TSPYL6	
LILRB3	
FAM101A	
LZTS1-AS1	
PDSS2	
LOC100129534	
ISLR2	
APOBEC4	
TPTE2P1	
MIR4791	
NAP1L3	
SNORD19B	
MIR5007	
ALDOA	
RPS7P5	
ATP6V1G1	
OR11H6	
KIR3DL2	

CLRN1-AS1	
ATL1	
OR1F2P	
PLA2G2E	
LINC00351	
FAM110A	
ZNF841	
MIR3193	
N4BP2L2	
CATSPERB	
MIR4762	
C17orf47	
TCHHL1	
NLRP14	
OR4S2	
LRCH3	
LALBA	
NDUFB8	
ZNF98	
IDH3G	
FAM24A	
OR6K2	
FOXR1	
GCG	
C20orf78	
GLS	
METTL20	
PRM3	
OR51B4	
MIR3662	
CES1P2	
MRGPRX2	
TLR7	
SCARNA9	
OR3A3	
SHBG	
CASKIN1	
OR1D4	
FAM71F2	
SLC10A1	
OR7C1	

MYO1H	
KIF11	
RFPL1S	
MIR450A1	
AGAP11	
SPO11	
ZMYND11	
MIR323B	
LOC649395	
KEL	
GP5	
LOC100288846	
MIR744	
GPR37	
CDC20B	
LRRC3C	
OR7G1	
SCNN1B	
MAP10	
PCP4	
LOC100289650	
LCA5L	
TOB2P1	
MAGI2	
SCN3A	
C4orf3	
GPR56	
OR8B12	
LINC00433	
SPINK5	
LINC00870	
GAPDHS	
MGC34034	
KCNA10	
ARMCX4	
SCRN2	
MSR1	
POLB	
LOC100129636	
ANXA2P1	
HIST1H1E	

FUZ	
HTR3E	
LOC729950	
CRYZ	
S1PR4	
NAE1	
GIMAP1-GIMAP5	
TRPV3	
FAM83A	
COL6A6	
SLC35E3	
MIR4669	
BMS1P1	
GSTO2	
PLVAP	
WDR75	
MIR3677	
C22orf46	
PSMC6	
CYP2W1	
SGK2	
MIR376B	
NMUR2	
OR13C4	
B3GALT5	
KLHDC4	
MIR4697HG	
HHAT	
SYCE2	
LRIT3	
GPRC5C	
CEMP1	
MIR4301	
MIR199A1	
HEMK1	
MIR583	
LRRTM2	
MIR4699	
YWHAQ	
RNF133	
CHIAP2	

ARHGEF3-AS1	
SNORD63	
MIR4264	
RAB30	
ANKRD37	
SIGLEC10	
TARDBP	
GPR115	
SALL3	
SNORD115-17	
MIR450A2	
MIR593	
ADCYAP1	
HNRNPU-AS1	
TGFBR1	
MIR331	
FAM26E	
UTRN	
PITPNM2	
S100A7L2	
HIST1H2AJ	
HS3ST1	
SUN1	
MIR1973	
TSIX	
MIR539	
KLRG1	
CRCT1	
LOC646268	
WFDC13	
OR5K1	
MIR135A2	
CLEC10A	
LCE2D	
ZNF577	
MIR10B	
C16orf90	
FPR2	
AA06	
MTMR8	
MIR654	

LINC00906	
MTRNR2L3	
WFDC10A	
ATP13A5	
ZMAT4	
PXDNL	
LOC441666	
SIGLECL1	
LOC285000	
LOC100289473	
ZFYVE20	
OR2M2	
C14orf180	
FUT2	
SELL	
LNX1-AS2	
ZNF876P	
TNFAIP8L2	
LOC100507346	
MIRLET7A2	
PIPSL	
TCF20	
MIR655	
FAM83B	
KIF4B	
PSMB11	
C9orf170	
KIF24	
LOC100130357	
MIR3607	
SPATA13-AS1	
OR11L1	
CYP2C19	
MIR3131	
MIR378C	
F2RL2	
LCT	
PIN4	
RASA4CP	
C3orf79	
LINC00304	

SNORD116-15	
LOC100287834	
LAIR1	
LINC00251	
NXPE2	
RILPL2	
MAD2L1	
UNC45A	
HRH4	
SNORA8	
SNORD30	
TRIM49B	
XIRP2	
CALU	
CYCSP52	
STH	
MIR4694	
LOC158435	
ROS1	
METTL21C	
FAAH	
SLC25A27	
LRRN4	
CBX5	
MTRF1	
CDH19	
FABP2	
RSPO2	
SLC47A2	
SNORD97	
XIAP	
IL23R	
SIRPD	
RNF216-IT1	
IL12RB2	
MIR4776-2	
KRTAP20-3	
MIR4776-1	
XCR1	
NXF4	
SNORD115-32	

DPF2	
OR5D18	
DYNAP	
MIR2355	
TRO	
PYGM	
TEK	
MIR548AC	
CD1D	
FCN1	
MIR376A2	
PRICKLE2-AS3	
PDGFA	
CHST4	
LINC00277	
CHST9	
SGCB	
TEX33	
ABCC12	
MIR891B	
CSMD3	
LOC285740	
MYOF	
MIR5704	
TFEC	
DBH	
COG2	
LOC100505875	
RNASE12	
FAM47E	
FMO1	
STXBP5L	
CDH20	
CFTR	
C14orf183	
SMIM2-IT1	
CCDC141	
PRSS53	
TSSC1	
RARB	
MYLK4	

MIR320D2	
PZP	
FLJ22447	
S100Z	
FDXACB1	
LOC286370	
MIR4705	
CLSTN2	
RNASE1	
CYP21A2	
LCP1	
IDO2	
ERP27	
SLC18A1	
MRAP	
PIGR	
MYL5	
MIR4282	
ABCC6P1	
SNORD21	
MIR20B	
LINC00880	
MIR3975	
C10orf71	
DMC1	
SNORA56	
LOC649330	
CABIN1	
PIK3C2B	
DHX58	
C7orf65	
PLD4	
TOP3B	
CRNKL1	
FAM179B	
HCG4B	
MIR554	
ZNF134	
ENTPD5	
GMNC	
CPA2	

TECRL	
CCDC38	
BTN2A2	
CXCL13	
TEX28	
RABL2B	
PILRB	
FPR1	
CYP2C9	
LINC00538	
MAGT1	
HMGCLL1	
MIR4441	
NYX	
ADRA2C	

Supp. Table 2. The 23 ENCODE datasets used for regulatory factor binding sites in Figure 6 and Supp. Fig. 3 (2011).

Regulatory factor	ENCODE dataset
ATF3	wgEncodeAwgTfbsHaibA549Atf3V0422111Etoh02UniPk.narrowPeak
BCL3	wgEncodeAwgTfbsHaibA549Bcl3V0422111Etoh02UniPk.narrowPeak
BHLHE40	wgEncodeAwgTfbsSydhA549Bhlhe40IggrabUniPk.narrowPeak
c-Myc	wgEncodeSydhTfbsA549CmycIggrabPk.narrowPeak
CEBP1	wgEncodeSydhTfbsA549CebpbIggrabPk.narrowPeak
CREB1	wgEncodeAwgTfbsHaibA549Creb1sc240V0416102Dex100nmUniPk.narrowPeak
CTCF	wgEncodeAwgTfbsHaibA549Ctcfsc5916Pcr1xEtoh02UniPk.narrowPeak
ELF1	wgEncodeAwgTfbsHaibA549Elf1V0422111Etoh02UniPk.narrowPeak
ETS1	wgEncodeAwgTfbsHaibA549Ets1V0422111Etoh02UniPk.narrowPeak
FOSL2	wgEncodeAwgTfbsHaibA549Fosl2V0422111Etoh02UniPk.narrowPeak
FOXA1	wgEncodeAwgTfbsHaibA549Foxa1V0416102Dex100nmUniPk.narrowPeak
GABPA	wgEncodeAwgTfbsHaibA549GabpV0422111Etoh02UniPk.narrowPeak
MAX	wgEncodeAwgTfbsSydhA549MaxIggrabUniPk.narrowPeak
NRSF	wgEncodeAwgTfbsHaibA549NrsfV0422111Etoh02UniPk.narrowPeak
p300	wgEncodeAwgTfbsHaibA549P300V0422111Etoh02UniPk.narrowPeak
Pol II	wgEncodeAwgTfbsHaibA549Pol2Pcr2xEtoh02UniPk.narrowPeak
RAD21	encode/wgEncodeAwgTfbsSydhA549Rad21IggrabUniPk.narrowPeak
SIN3A	wgEncodeAwgTfbsHaibA549Sin3ak20V0422111Etoh02UniPk.narrowPeak
SIX5	wgEncodeAwgTfbsHaibA549Six5V0422111Etoh02UniPk.narrowPeak
TAF1	wgEncodeAwgTfbsHaibA549Taf1V0422111Etoh02UniPk.narrowPeak
TCF	wgEncodeAwgTfbsHaibA549Tcf12V0422111Etoh02UniPk.narrowPeak
YY1	wgEncodeAwgTfbsHaibA549Yy1cV0422111Etoh02UniPk.narrowPeak
ZBTB33	wgEncodeAwgTfbsHaibA549Zbtb33V0422111Etoh02UniPk.narrowPeak

Supp. Table 3. Global correlation values for iSLK.219 cell line 0 hour with the 6, 12, 24, and 48 hours post doxycycline treatment and corresponding timepoints for control iSLK.219 cells.

			Doxycycline treated timepoints				Corresponding control timepoints			
		0 HR	6 HR	12 HR	24 HR	48 HR	6 HR	12 HR	24 HR	48 HR
	0 HR	1	0.97489037	0.96960797	0.84697906	0.93947887	0.97564998	0.96205344	0.9352317	0.95823614
Doxycycline treated timepoints	6 HR	0.97489037	1	0.97405963	0.84593782	0.93957052	0.97913783	0.9611058	0.93228324	0.95685885
	12 HR	0.96960797	0.97405963	1	0.87916155	0.96029981	0.97984215	0.96192273	0.95514304	0.9647646
	24 HR	0.84697906	0.84593782	0.87916155	1	0.90944492	0.86380829	0.85352642	0.90773688	0.89952851
	48 HR	0.93947887	0.93957052	0.96029981	0.90944492	1	0.95119799	0.93854611	0.95728881	0.96055785
Corresponding control timepoints	6 HR	0.97564998	0.97913783	0.97984215	0.86380829	0.95119799	1	0.96637524	0.94921343	0.96528891
	12 HR	0.96205344	0.9611058	0.96192273	0.85352642	0.93854611	0.96637524	1	0.93897712	0.95275119
	24 HR	0.9352317	0.93228324	0.95514304	0.90773688	0.95728881	0.94921343	0.93897712	1	0.95906846
	48 HR	0.95823614	0.95685885	0.9647646	0.89952851	0.96055785	0.96528891	0.95275119	0.95906846	1

Supp. Table 4. Number of raw reads, aligned reads to HG19 genome, on- and off-target reads for 0, 6, 12, 24, and 48 hours for iSLK.219 doxycyline treated and corresponding timepoints for control iSLK.219 cells.

		Raw reads	Aligned reads	On-target reads	Off-target reads	Percent on-target reads	Percent off-target reads
Doxycycline treated timepoints	6HR	28,818,960	26,345,075	24,253,601	2,091,474	92.06%	7.94%
	12HR	21,946,955	20,121,373	18,463,933	1,657,440	91.76%	8.24%
	24HR	8,531,342	7,797,542	7,159,867	637,675	91.82%	8.18%
	48HR	16,165,852	14,737,424	13,397,681	1,339,743	90.91%	9.09%
Corresponding control timepoints	0HR	20,458,718	18,658,368	17,228,108	1,430,260	92.33%	7.67%
	6HR	30,184,600	27,620,979	25,368,509	2,252,470	91.85%	8.15%
	12HR	13,357,715	12,139,566	11,120,008	1,019,558	91.60%	8.40%
	24HR	9,053,492	8,278,466	7,569,039	709,427	91.43%	8.57%
	48HR	15,389,683	14,037,853	12,855,496	1,182,357	91.58%	8.42%
